# β-Branching in the biosynthesis of bongkrekic acid: a complex affair

**DOI:** 10.1039/d5ra05400a

**Published:** 2025-10-28

**Authors:** Megan E. M. Hiseman, Annabel P. Phillips, Ciprian Chiriac, Liam J. Smith, John Crosby, Christopher Williams, Christine L. Willis, Ashley J. Winter, Matthew P. Crump

**Affiliations:** a School of Chemistry, University of Bristol Bristol BS8 1TS UK ash.winter@bristol.ac.uk matt.crump@bristol.ac.uk

## Abstract

Bongkrekic acid is a potent respiratory toxin which inhibits the mitochondrial ATP/ADP carrier protein. The polyketide synthase that biosynthesises bongkrekic acid recruits a discrete cassette of β-branching enzymes (BonF–BonI) to install two distinct β-branches: an *endo*-β-methyl branch in module 1, and a carboxymethyl β-branch in module 11. Both β-branches contribute to specific interactions with bongkrekic acid's biological target. However, a critical component of the β-branching cassette, the donor acyl carrier protein (ACP_D_), has not been identified in previous studies. Furthermore for the module 11 carboxymethyl β-branch to be retained, conversion to an *endo*-β-methyl branch *via* the enoyl-coenzyme A hydratase (ECH), BonI, must be avoided. The mechanistic basis for these divergent β-branching pathways is poorly understood, both in the bongkrekic acid biosynthetic pathway and more generally where it arises in polyketide biosynthesis. Here, we confirm the roles of BonF–BonI by reconstituting β-branching in modules 1 and 11 *in vitro* and uncover the previously unannotated ACP_D_, BonN, to complete the β-branching cassette. We further demonstrate promiscuous BonI interactions with both module 1 and 11 ACPs that confounds simple ACP selectivity arguments for carboxymethyl β-branch *versus endo*-β-methyl branch installation, suggesting that this is instead regulated by a complex interplay between substrate and kinetic control.

## Abstract 2

Bongkrekic acid biosynthesis requires a cassette of β-branching enzymes (BonF–BonI) to install two distinct β-branches. Here we confirm the roles of BonF–BonI *in vitro* and uncover a previously unannotated ACP_D_, BonN.

## Graphical abstract

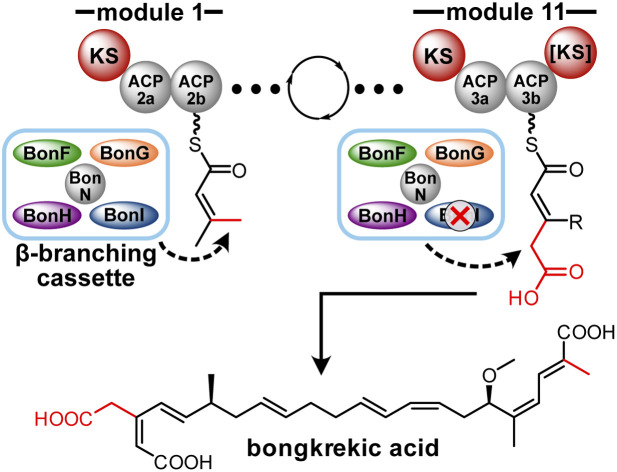

## 1 Introduction

Polyketides are a class of natural products that are widely recognised for their valuable bioactivities, including antibacterial, antifungal and anticancer properties.^1,2^ Whilst their structures are often complex, they share common basic biosynthetic machinery to create a functionalised carbon chain (the assembly phase). This occurs on an acyl carrier protein (ACP) modified with a flexible phosphopantetheine (Ppant) arm that tethers the substrate. The ACP is primed with acyl-coenzyme A (CoA) building blocks, such as malonyl-CoA, *via* the action of an acyltransferase (AT), and a ketosynthase (KS) extends the polyketide chain *via* decarboxylative Claisen condensation.^3,4^ The recursive use of these functions results in chain elongation, but at each stage of carbon extension, the ACP-bound β-ketothioester can be further modified through the action of specific enzyme domains that results in chemical, structural and functional diversification. In type I polyketide synthases (PKSs), the catalytic domains are typically organised into one or more modules encoded by a single polypeptide and the polyketide is processed and shuttled along the PKS whilst tethered to successive ACPs.^5^ Further chemical diversity may be introduced by the presence of additional *cis*- or *trans*-acting enzymes, such as monooxygenases, methyltransferases and halogenases.^6^ The presence of *cis*-AT domains *versus trans*-AT domains, for example, is a distinguishing feature of the two major classes of type I PKSs.^6^

**Fig. 1:**
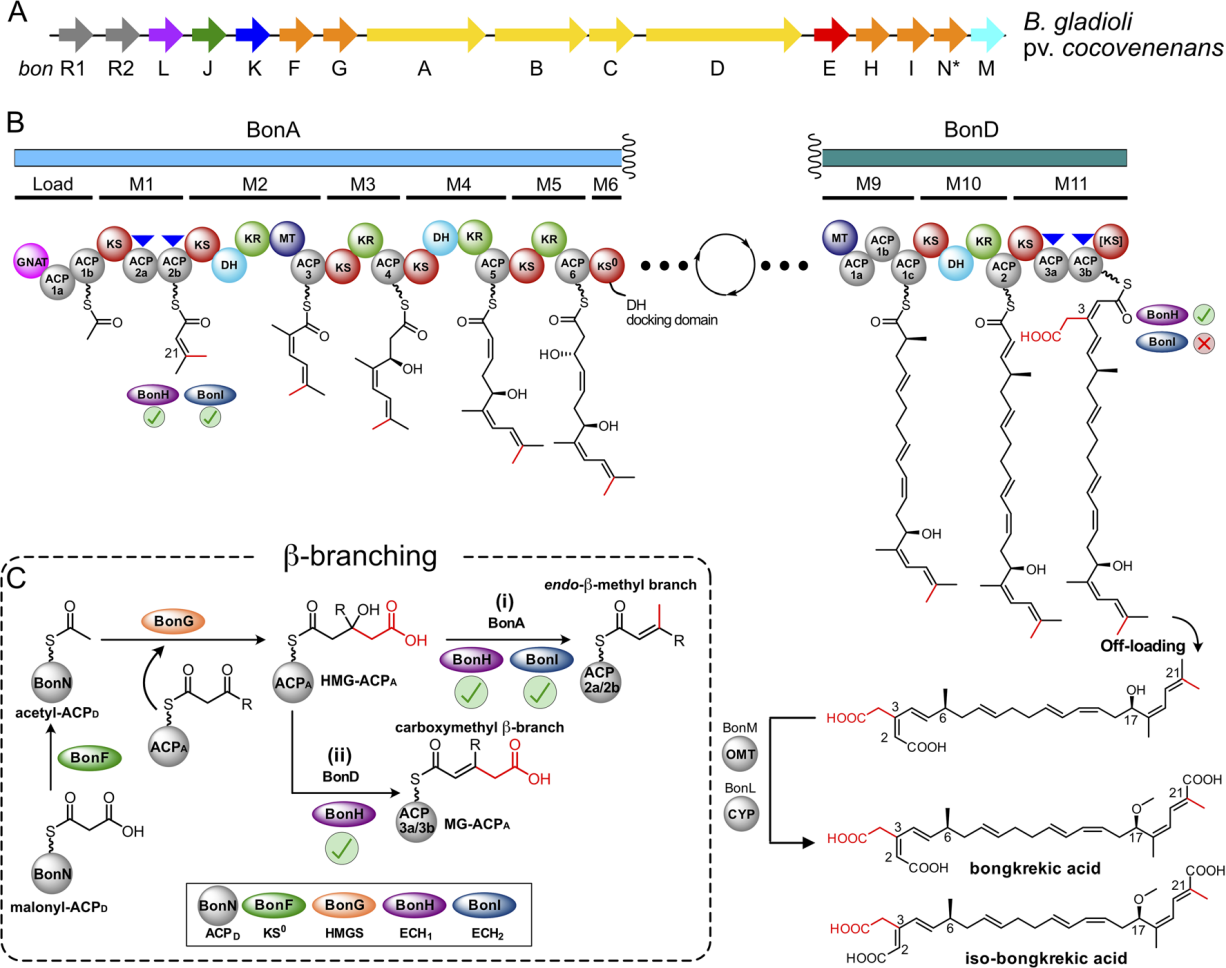
Overview of bongkrekic acid biosynthesis. (A) *Burkholderia gladioli* pv. *cocovenenans bon* biosynthetic gene cluster (BGC). Genes are highlighted as follows: regulatory (grey), enoyl reductase (red), acyl hydrolase (green), acyltransferase (dark blue), β-branching cassette enzymes (orange), type I polyketide synthase (PKS, yellow), *O*-methyltransferase (OMT, light blue) and cytochrome P450 (CYP, purple). The newly discovered BonN is marked with an asterisk (this study). (B) Bongkrekic acid biosynthetic pathway. Type I PKS BonA and BonD incorporate β-branches at acceptor ACPs (ACP_A_s, blue arrows). β-Branches are shown in red. (C) Proposed divergent branch formation between modules BonA (i) and BonD (ii) to incorporate an *endo*-β-methyl branch and a carboxymethyl β-branch (both highlighted in red). In the context of an advanced polyketide intermediate, the carboxymethyl β-branch is equivalent to an MG-intermediate. The stereochemistry of the carboxylated β-branched intermediate is shown *cis* to the alkene proton of the β-ketothioester, with the polyketide intermediate *trans*. A similar bond rotation upon decarboxylation to form the endo β-branch is assumed as shown by Walker *et al.*^9^ (inset) HMGS cassette components.

β-Branching is a notable example of polyketide structural diversification prevalent in *trans*-AT PKSs and installation of this alkyl moiety has been shown to be important for bioactivity.^7,8^ The alkylation of a post-decarboxylative Claisen condensation β-keto group is carried out by a series of discrete proteins known as a 3-hydroxy-3-methylglutaryl synthase (HMGS) cassette. Typically, a malonyl unit bound to a distinct ACP is first decarboxylated by a non-elongating ketosynthase (KS^0^). The discrete ACP donates the resulting acetyl unit to the HMGS for subsequent aldol addition with the polyketide β-ketothioester, and is therefore referred to as a donor ACP (ACP_D_). The ACP that tethers the polyketide β-ketothioester during β-branching is referred to as the acceptor (ACP_A_). Following aldol addition, the 3-hydroxy-3-methylglutaryl (HMG)-like product is then dehydrated in a reversible equilibrium by an enoyl-CoA hydratase (ECH_1_) to produce a transient 3-methylglutaconyl (MG)-ACP_A_ analogue (Fig. 1).^8^ The ACP_D_, KS^0^, HMGS and ECH_1_ form the minimal HMGS cassette components though several known HMGS cassettes, such as those employed in the kalimantacin and mupirocin biosynthetic pathways, also encode a decarboxylating enoyl-CoA hydratase (ECH_2_) that can form an α,β- or β,γ-unsaturated methyl branch, depending on its regioselectivity.^9,10^ In contrast, the bryostatin and leinamycin biosynthetic pathways lack a *trans*-acting ECH_2_, which leads to retention of a carboxylated β-branch.^11,12^ The incorporation of multiple classes of β-branches within a single polyketide is governed by precise regulatory mechanisms and often requires duplicate HMGS cassette components with divergent ACP_A_ selectivity (*e.g.*, kalimantacin and myxovirescin biosynthesis).^7^

Bongkrekic acid is a respiratory toxin isolated from multiple *Burkholderia gladioli* strains and is an example of a polyketide with more than one distinct β-branch (Fig. 1).^13,14^ The chemical structure comprises a polyunsaturated carbon backbone with three terminal carboxylic acids, one of which is a β-branch. Bongkrekic acid is the major metabolite produced by *B. gladioli*, and its toxicity is due to inhibition of the mitochondrial ATP/ADP carrier, a function linked to its tricarboxylic acid structure.^15^ Iso-bongkrekic acid, a minor metabolite (<10% yield of bongkrekic acid) has also been isolated from *B. gladioli* pathovar (pv.) *cocovenenans* with a lower toxicity resulting from a 2- to 4-fold decrease in inhibition of the ATP/ADP carrier.^16,17^ The bongkrekic acid core PKS consists of four major genes that between them encode a loading domain and 11 extension modules (Fig. 1A and B). An α,β-unsaturated methyl branch (referred to as an *endo*-β-methyl branch) at C-21 is installed in BonA module 1, whereas the C-3 carboxymethyl β-branch is introduced in the final module (BonD module 11). Both β-branches contribute to the specific interactions of bongkrekic acid with its biological target.^18^ The 2,3-alkene stereochemistry (*Z*-isomer for bongkrekic acid, *E*-isomer for iso-bongkrekic acid) introduced by installation of the C-3 β-branch is the only structural difference between the two congeners. Four *trans*-acting HMGS cassette components have been identified in the *bon* biosynthetic gene cluster (BGC) to-date: BonF (KS^0^), BonG (HMGS), BonH (ECH_1_) and BonI (ECH_2_) (Fig. 1C). Several important biosynthetic features, however, remain elusive. A gene encoding a candidate *trans*-acting ACP_D_ for example, has not been identified in any characterised *bon* BGCs so far (Fig. 1A). Additionally, all HMGS components are required to generate the *endo*-β-methyl moiety at the beginning of the biosynthetic pathway in module 1 but it remains unclear how the subsequent carboxymethyl β-branch introduced in the terminal module 11 escapes the decarboxylative action of the *trans*-acting ECH_2_, BonI. One possibility is that control is exerted by strict ACP_A_/BonI specificity that excludes interaction with the late-stage ACP_A_, however, this remains unconfirmed.

Studies presented here integrate bioinformatic analyses, recombinant purified proteins and *in vitro* mass spectrometry assays to identify a conserved discrete ACP and confirm its role as an ACP_D_. Reconstitution of HMGS cassette enzymes and derivatised acceptor ACPs from two modules (BonA and BonD ACP_A_s) has also revealed the surprising promiscuity of key HMGS components and provides new insights into the control mechanisms that may regulate β-branching in the bongkrekic acid biosynthetic pathway.

## 2 Results and discussion

Identification of a putative ACP_D_ began with reanalysis of the *bon* BGC from *Burkholderia gladioli* pv. *cocovenenans* (NCBI accession: JX173632.1) using antiSMASH 7.0.^19^ Initially, genes encoding discrete candidate ACP_D_s could not be identified, but antiSMASH uncovered two ACP didomains, one in the loading module (BonA_ACP1a-1b, 63.5% sequence identity) and one in module 1 (BonA_ACP2a-2b, 98% sequence identity) in contrast to single ACP domains previously annotated (BonA_ACP1 and BonA_ACP2) (Fig. 1 and S1).^13^ Both sets of ACPs appeared to be tandem domains and assumed to have equivalent function based on their high sequence conservation. However, neither of these domains were potential ACP_D_s. As an alternate route to identifying a candidate ACP_D_, the amino acid sequence of the ACP_D_ encoded by the gladiolin BGC from *B. gladioli* BCC0238, GbnF (NCBI accession: WP_036053932.1), was used as a probe for Protein BLAST database searches.^20^ Searches were conducted against *B. gladioli* pv. *cocovenenans* and a homologous strain, *B. gladioli* BSR3 (*bon* BGC amino acid sequence identity of 98–99%).^21^ As a result, eight ACP candidates with sequence identities greater than 50% were identified (Table S1). A manual search of all *bon* BGCs was then carried out using each of the candidate ACP_D_ sequences identified by Protein BLAST. When the amino acid sequence for WP_013698117 was used as a probe (59% sequence identity with GbnF), a gene encoding this ACP with 100% conservation was found in the intergenic region between *bon*I (ECH_2_) and *bon*M (*O*-methyltransferase) (Fig. 1A). A gene encoding this exact amino acid sequence was identified in the same position in the *B. gladioli* BSR3 BGC (Fig. S2) and also identified in an additional 32 *bon* BGCs across *B. gladioli* genomes (Fig. S3).^14^

To determine if the newly identified ACP (henceforth termed BonN) resembled an ACP_D_, a maximum-likelihood phylogenetic tree was generated (Fig. S4) using 51 ACP_D_ amino acid sequences from characterised *cis*- and *trans*-AT PKSs, including BonN. 14 modular ACPs from the BonA–BonD PKSs were included to rule out the unlikely possibility of a *cis*-acting ACP_D_. The ACP_D_s and *cis*-acting Bon ACPs form two distinct clades, with BonN clustering specifically with HamF and PulM, which are part of the biosynthetic pathways for hamuramicin C and pulvomycin respectively. These two ACP_D_s utilise an acetyl unit for HMGS-catalysed aldol addition, and clade separately from ACP_D_s that employ methylmalonyl/propionyl units.^22,23^ As both β-branches installed in the bongkrekic acid biosynthetic pathway are derived from an acetyl donor, the bioinformatic analyses suggested BonN to be a candidate ACP_D_. Conversely, both newly identified tandem ACP domains did not clade with the ACP_D_ species.

**Fig. 2:**
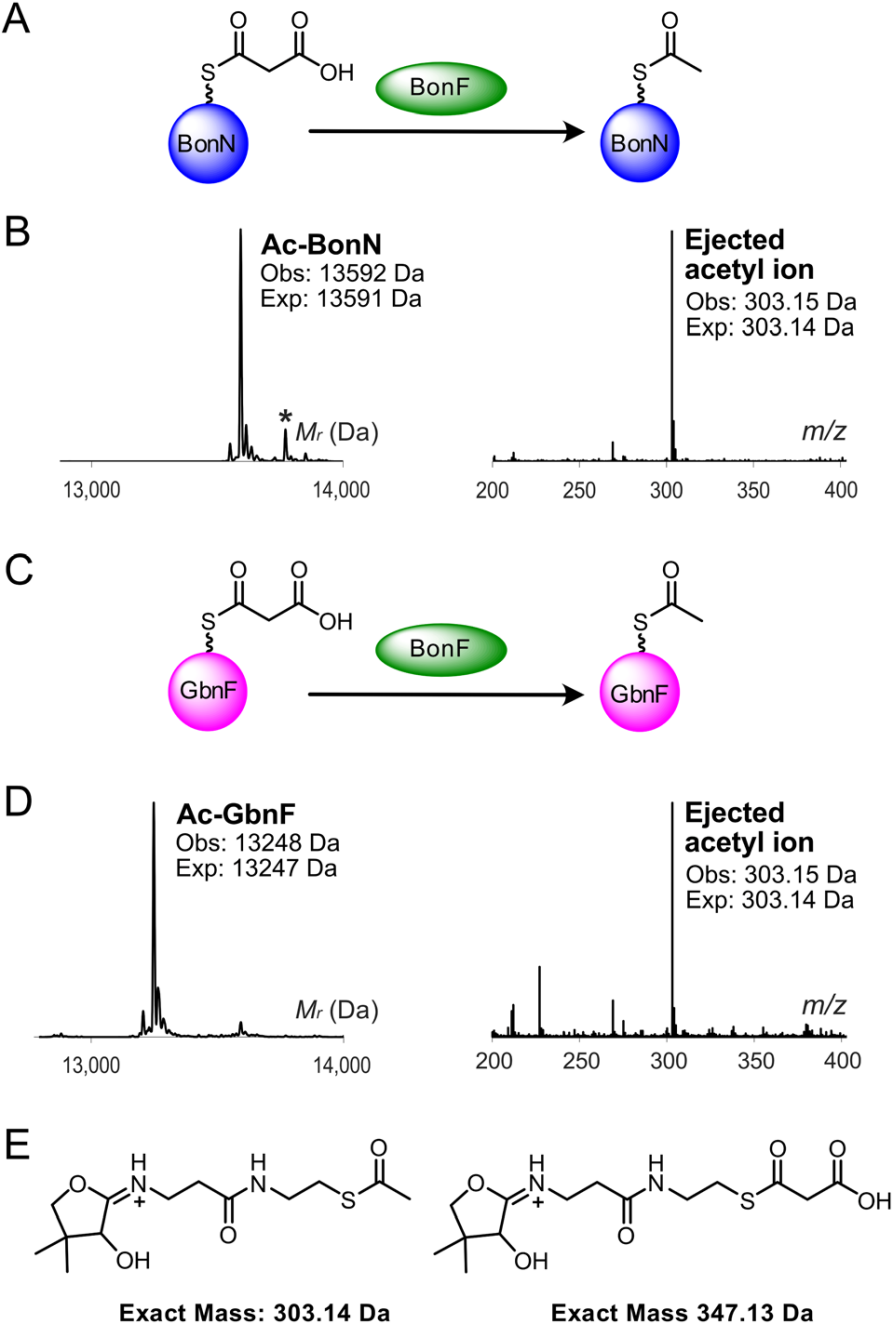
BonF-catalysed decarboxylation of malonyl-BonN. (A) Proposed reaction scheme for BonF-catalysed decarboxylation of malonyl-BonN. (B) Deconvoluted spectrum and corresponding Ppant ejection of BonF assay with malonyl-BonN. * refers to phosphogluconoylation of the His6 tag (+178 Da).^26^ (C) Scheme for BonF-catalysed malonyl-GbnF decarboxylation. (D) Deconvoluted spectrum and corresponding Ppant ejection of BonF assay with malonyl-GbnF. (E) Expected Ppant ejection ions.

To confirm the function of BonN as the missing β-branching ACP_D_, its involvement in two critical steps, malonyl-ACP_D_ decarboxylation and aldol addition, was tested. We cloned, expressed and purified BonN and the homologous putative ACP_D_, GbnF (Fig. S5 and Table 1). Both ACPs were purified in their *apo* form, and analytical size exclusion chromatography (SEC) suggested they were both monomeric in solution. Analysis by ^1^H nuclear magnetic resonance (NMR) spectroscopy and circular dichroism (CD) determined that both discrete ACPs were folded and predominantly α-helical in structure. We also overproduced and purified the *trans*-acting KS^0^ BonF, which is responsible for decarboxylating a malonyl-ACP_D_ species to form acetyl-ACP_D_, prior to HMGS-catalysed aldol addition. Analytical SEC confirmed BonF was monomeric in solution (Fig. S5). *apo*-BonN and *apo*-GbnF were converted to their malonyl-ACP derivatives (malonyl-BonN (obs: 13 635 Da, exp: 13 636 Da) and malonyl-GbnF (obs: 13 292 Da, exp: 13 292 Da)) using malonyl-CoA and the promiscuous phosphopantetheinyl transferase, MupN (Fig. S6).^24^ Malonyl-BonN was first incubated with BonF and the decarboxylation reaction monitored by electrospray mass spectrometry (ESMS). This confirmed that the malonyl-ACP was recognised and decarboxylated by BonF to yield acetyl-BonN (obs: 13 592 Da, exp: 13 591 Da) (Fig. 2A and B). Ppant ejection assays *via* collision-induced dissociation of the derivatised Ppant arm were used to provide characteristic fragmentation ions for high mass accuracy detection of low molecular weight intermediates.^25^ Ppant ejection produced the expected acetylated ion (obs: 303.15 Da, exp: 303.14 Da).

**Table 1:** Constructs used within this study and their role

Construct	Role
GbnF	Donor ACP encoded in gladiolin biosynthesis
BonN	Donor ACP encoded in bongkrekic acid biosynthesis
BonF	KS^0^ responsible for decarboxylation of the malonyl-donor ACP to form an acetyl-ACP
BonA_ACP1a	ACP encoded in loading module of BonA
BonD_ACP1b	ACP assumed to be involved in α-methylation in BonD module 9
BonD_ACP3b	Acceptor ACP encoded in BonD module 11
BonG	HMGS responsible for aldol addition with the β-ketothioester bound to an acceptor ACP to form HMG-ACP
BonA_ACP2a	Acceptor ACP encoded in BonA module 1
BonH	ECH_1_ catalysing dehydration of HMG-ACP to form MG-ACP (the carboxylated β-branch at C3)
BonI	ECH_2_ responsible for decarboxylation of MG-ACP to form the *endo*-β-methyl branch at C21
MupN	Promiscuous phosphopantetheinyl transferase involved in mupirocin biosynthesis

BonF could also recognise the homologous ACP_D_, GbnF, decarboxylating malonyl-GbnF to acetyl-GbnF (obs: 13 248 Da, exp: 13 247 Da) (Fig. 2C and D). Control experiments using denatured BonF resulted in no observable decarboxylation of malonyl-BonN and malonyl-GbnF, confirming the catalytic role of this enzyme (Fig. S7A and B).

To determine if BonF was a promiscuous KS^0^ or displayed specific activity for ACP_D_s, we expressed and purified three modular ACPs: BonA_ACP1a (load module), BonD_ACP1b (module 9) and BonD_ACP3b (module 11) (Fig. S8). Each excised modular ACP was soluble and confirmed to be monomeric in solution by analytical SEC prior to chemoenzymatic derivatisation to produce malonyl-ACPs (Fig. S6). Individual incubation of malonylated ACPs with BonF showed that BonF could not decarboxylate malonyl-BonA_ACP1a, malonyl-BonD_ACP1b and malonyl-BonD_ACP3b (Fig. S7). These results indicate that BonF specifically recognises BonN.

Next, we expressed and purified the HMGS, BonG, to reconstitute aldol addition *in vitro* (Fig. S5). For representative ACP_A_ components, we initially selected the BonD_ACP3a-3b ACP_A_ didomain from module 11.^13^ The ACPs share 76% sequence identity, and both contain a characteristic tryptophan flag, a common recognition motif for HMGS cassette components, suggesting that ACP3a and ACP3b act in-tandem (Fig. S1). When overexpressed, however, all attempts to produce BonD_ACP3a yielded insoluble protein (data not shown). In contrast, expression and purification of the second ACP, BonD_ACP3b, was successful (Fig. S8).

**Fig. 3:**
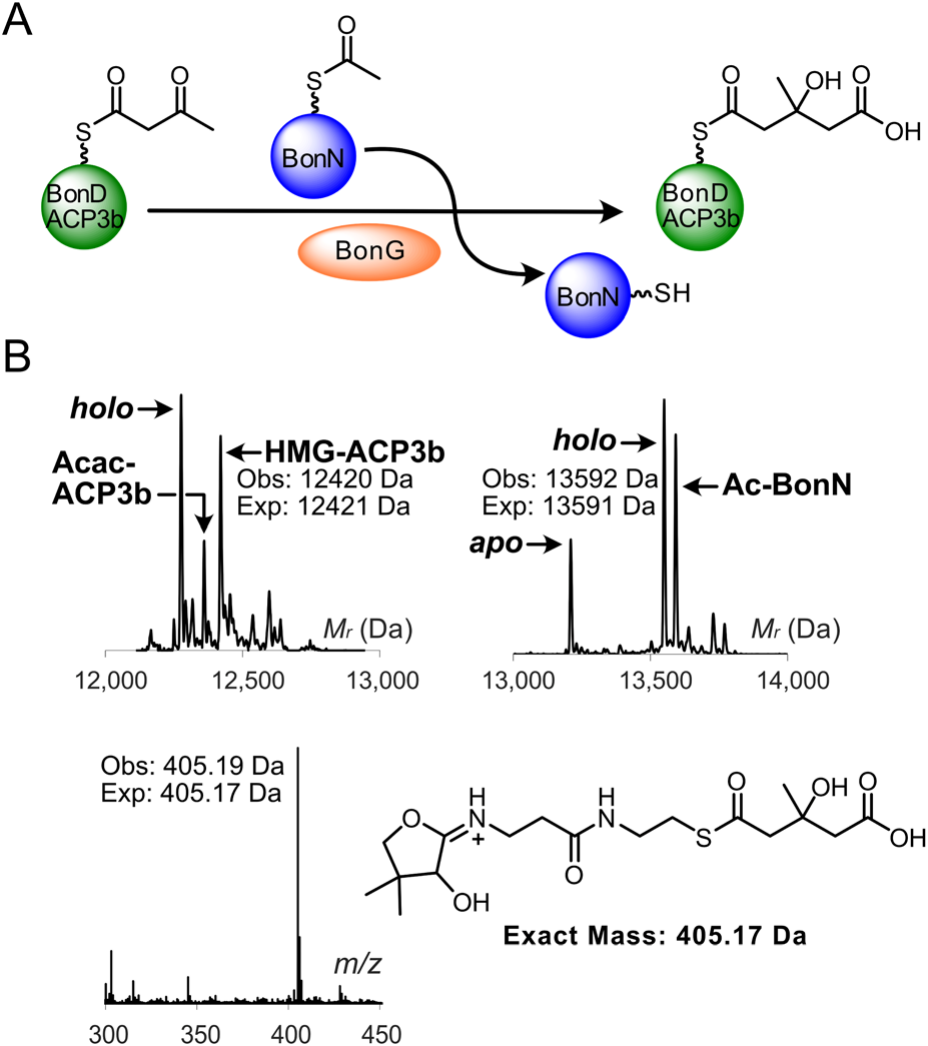
BonG-catalysed aldol addition scheme and ESMS assays. (A) Proposed reaction scheme for BonG-catalysed aldol addition of acetoacetyl (Acac)-BonD_ACP3b with acetyl (Ac)-BonN. (B) Deconvoluted spectrum and corresponding Ppant ejection of BonG assay with Acac-BonD_ACP3b and (Ac)-BonN. (Inset) Expected Ppant ejection ion for the HMG species.

Acetyl-BonN and acetoacetyl-BonD_ACP3b were initially generated chemoenzymatically (Fig. S9)^9,27^ and then incubated with BonG (Fig. 3A). Reaction monitoring by ESMS revealed a new ACP3b-bound species with a mass consistent with HMG-BonD_ACP3b (obs: 12 420 Da, exp: 12 421 Da) (Fig. 3B). Ppant ejection produced a characteristic HMG-Ppant fragmentation ion (obs: 405.19 Da, exp: 405.17 Da), verifying that BonG-catalysed aldol addition had occurred and confirming the role of BonN as the ACP_D_. As in previous HMGS-based assays, simultaneous hydrolysis of Ac-BonN to produce *holo*-BonN was also observed.^28,29^ Assays with denatured BonG abolished formation of HMG-BonD_ACP3b, confirming the function of the HMGS (Fig. S10). Substitution of acetyl-GbnF for acetyl-BonN also resulted in the formation of HMG-BonD_ACP3b, suggesting some flexibility in ACP_D_ recognition by BonG (Fig. S10).

To determine if BonG displayed a robust substrate specificity towards acetyl donor units, we chemoenzymatically derivatised BonN with propionyl-CoA (Fig. S11). No aldol addition with acetoacetyl-BonD_ACP3b was observed when propionyl-BonN was utilised in BonG HMGS assays (Fig. S11). We also reconstituted β-branching with the module 1 ACP_A_, BonA_ACP2a (98% sequence identity shared with its tandem ACP pair, BonA_ACP2b), which was expressed and purified as a discrete, monomeric ACP (Fig. S8). Incubation of BonG and acetyl-BonN with acetoacetyl-BonA_ACP2a also resulted in the formation of HMG-BonA_ACP2a (Fig. S12). However, BonG catalysed HMG formation was not observed when alternate ACP_A_ and ACP_D_ combinations were applied (Fig. S13–S15) confirming that strict molecular recognition governs ACP_D_*versus* ACP_A_ selection.^11,28^

**Fig. 4:**
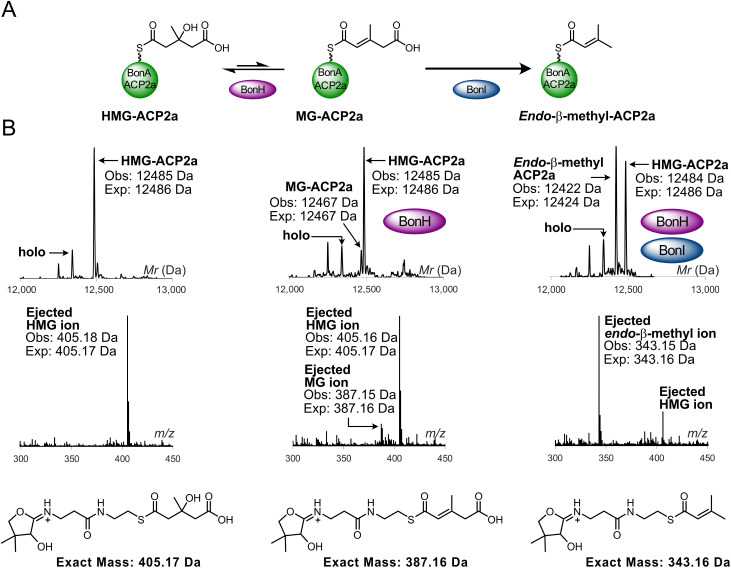
BonH- and BonI-catalysed dehydration and decarboxylation of HMG-BonA_ACP2a. (A) Reaction scheme to form *endo*-β-methyl-ACP2a from HMG-ACP2a *via* BonH and BonI, respectively. (B) Deconvoluted spectrum and corresponding Ppant ejection of HMG-ACP2a assays with BonH and BonI. (Inset) Expected Ppant ejection ion for HMG, MG and *endo*-β-methyl species.

To investigate whether the divergent incorporation of β-branches at module 1 *versus* module 11 is controlled by ACP specificity, we overproduced and purified both BonH (ECH_1_) and BonI (ECH_2_) to homogeneity (Fig. S16). Both ECHs were soluble and trimeric by analytical SEC.^30,31^ ACP/ECH recognition was first tested with BonA_ACP2a (Fig. 4A). Due to the transient nature of the MG-ACP_A_ intermediate, we derivatised BonA_ACP2a with (*R*,*S*)-HMG-CoA to simplify the assay and make detection of the MG intermediate more straightforward. Preparation of HMG-BonA_ACP2a was confirmed by ESMS (obs: 12 485 Da, exp: 12 486 Da) and Ppant ejection (obs: 405.18 Da, exp: 405.17 Da) (Fig. 4B).

HMG-BonA_ACP2a was initially incubated with BonH alone, and ESMS analysis confirmed BonH-catalysed dehydration to yield MG-BonA_ACP2a (obs: 12 467 Da, exp: 12 467 Da). This was verified by Ppant ejection, which generated the corresponding MG-Ppant ion (obs: 387.15 Da, exp: 387.16 Da). Addition of BonI resulted in the decarboxylation of MG-BonA_ACP2a, producing *endo*-β-methyl-BonA_ACP2a with total conversion of 50% of the HMG-BonA_ACP2a. This partial conversion is likely due to selection of a single stereoisomer of the ACP bound HMG (obs: 12 422 Da, exp: 12 424 Da). Ppant ejection analysis generated the expected *endo*-β-methyl Ppant ion (obs: 343.15 Da, exp: 343.16 Da), confirming that BonH and BonI functioned with a module 1 ACP_A_.

**Fig. 5:**
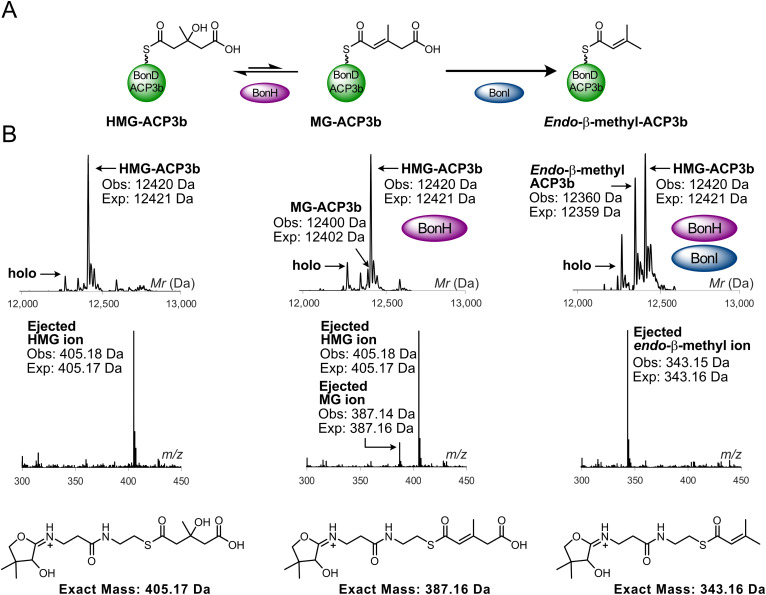
BonH- and BonI-catalysed dehydration and decarboxylation of HMG-BonD_ACP3b. (A) Reaction scheme whereby BonI converts MG-ACP3b to *endo*-β-methyl-ACP3b instead of retaining the carboxylated β-branch as observed in the biosynthetic pathway. (B) Deconvoluted spectrum and corresponding Ppant ejection of HMG-ACP3b assays with BonH and BonI. (Inset) Expected Ppant ejection ion for HMG, MG and *endo*-β-methyl species.

To test if a module 11 ACP_A_ recognises BonH, but rejects BonI (Fig. 5A), BonD_ACP3b was selected as the ACP_A_ and HMG-BonD_ACP3b prepared as described for BonA_ACP2a (obs: 12 420 Da, exp: 12 421 Da) (Fig. 5B). Incubation with BonH resulted in a product with a mass corresponding to MG-BonD_ACP3b (obs: 12 400 Da, exp: 12 402 Da) that gave the correct MG Ppant ejection ion (obs: 387.14 Da, exp: 387.16 Da). Upon incubation of HMG-BonD_ACP3b with BonH and BonI, the MG species was, surprisingly, efficiently converted to *endo*-β-methyl-BonD_ACP3b (obs: 12 360 Da, exp: 12 359 Da). This result was confirmed by Ppant ejection analysis, which yielded the characteristic *endo*-β-methyl Ppant ion (obs: 343.15 Da, exp: 343.16 Da). This suggested that BonI could in fact work in tandem with BonH and convert its MG-BonD_ACP3b product to the incorrect *endo*-β-methyl branch. BonI exclusion is therefore not controlled by ACP specificity.

To determine if the Bon ECHs, and particularly BonI, displayed more general ACP promiscuity, we derivatised non-acceptors BonA_ACP1a and BonD_ACP1b with HMG (Fig. S17 and S18). BonH and BonI were capable of converting HMG-ACP species to form MG-ACP products and *endo*-β-methyl-ACP products, albeit only in small amounts (15%) for BonA_ACP1a. Interestingly, these findings suggest that both BonH and BonI exhibit notable ACP promiscuity. In these specific cases, however, this promiscuity does not pose a biosynthetic concern, as the ACPs involved are not capable of forming the HMG intermediate under native conditions.

In this study, we have identified BonN, a previously unannotated ACP within the *bon* BGCs, have firmly established its role as an ACP_D_, and have fully reconstituted the β-branching pathway in bongkrekic acid biosynthesis. We have demonstrated that BonN functions as an ACP_D_ in conjunction with BonF (KS^0^) and BonG (HMGS) across modules 1 and 11, facilitating the installation of the C-21 and C-3 β-branches, respectively. This establishes BonN as an essential component of the HMGS cassette.

Introduction of the C-3 carboxymethyl β-branch within module 11 is essential for the biological activity of bongkrekic acid, which mimics ATP to inhibit the mitochondrial ATP/ADP carrier *via* its tricarboxylic structure.^15,17^ In the native system, retention of the C-3 carboxymethyl β-branch depends on the omission of a BonI catalysed decarboxylative step in module 11. *In vitro* reconstitution of β-branching on module 11 BonD_ACP3b using a simple (*R*,*S*)-HMG unit has shown that the ECH_2_, BonI, can however function with this ACP_A_. Furthermore, BonH and BonI can function with several non-ACP_A_s, highlighting significant promiscuity. Since ACP-bound HMG intermediates are required to investigate ECH promiscuity, their interaction with non-ACP_A_s has not been widely explored to the best of our knowledge. However, recent *in vitro* and *in vivo* studies of β-branching mechanisms in the virginiamycin biosynthetic pathway have suggested that HMGS cassette components can exhibit promiscuity and interact with non-β-branching ACPs.^31^ In contrast, BonF-catalysed malonyl decarboxylation and subsequent BonG-catalysed aldol addition reactions are selective, occurring only with their cognate β-branching ACPs. The high specificity of the HMGS components serves to prevent erroneous β-branch installation.

As *endo*-β-branch formation can occur on BonD_ACP3b (as a discrete domain), alternative control mechanisms must be employed within a modular context to prevent the action of BonI. The final biosynthetic β-branching step and polyketide off-loading may be coupled. Typically, the final polyketide intermediate is released from the PKS by a thioesterase (TE) domain. The *bon* BGC does not, however, encode a terminal TE domain or a candidate *trans*-acting enzyme. Instead, module 11 ends with an atypical ketosynthase domain (referred to as [KS] henceforth) which is conserved in *bon* BGCs.^13,14^ This [KS] lacks the conserved Cys–His–His catalytic triad characteristic of elongating KSs and is replaced by an unusual Ser–Ala–His triad which may facilitate polyketide chain release.^13,32–34^

**Fig. 6:**
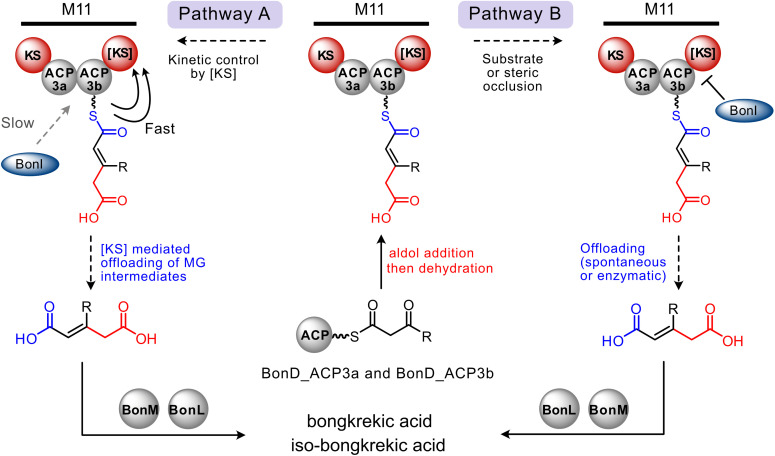
Proposed alternate pathways that may act in the final stages of bongkrekic acid biosynthesis to retain the carboxymethyl β-branch prior to substrate off-loading. After aldol addition and dehydration of the polyketide attached to BonD_ACP3a/b (red). Substrate off-loading (blue) could occur *via* kinetic control by the terminal [KS] domain (pathway A) or steric occlusion of BonI by the [KS] domain (pathway B), prior to off-loading. The *trans*-acting BonM and BonL furnish bongkrekic acid/iso-bongkrekic acid.

We hypothesise that the BonD [KS] may be intricately linked to off-loading of the final polyketide chain in bongkrekic acid biosynthesis *via* one of two proposed pathways (Fig. 6). Following formation of MG-ACP_A_ in module 11, BonI activity and polyketide off-loading could be under kinetic control (pathway A, Fig. 6). This may be achieved by faster in-*cis* chain release of the MG intermediate by the [KS] before BonI-mediated decarboxylation can occur. A similar kinetic control mechanism may control installation of a terminal carboxymethyl β-branch in the ripostatin biosynthetic pathway and, interestingly in this related example, a TE is present.^35^ Kinetic control is also hypothesised to govern β-branching in the kalimantacin biosynthetic pathway.^9^ Here, in module 11, in-*cis* substrate channelling *via* a modular ECH_2_ (mECH) must out-compete recruitment of a *trans*-acting ECH_2_ (BatE) to generate an alternate *exo*-β-methylene branch. This in-*cis versus* in-*trans* control may also govern β-branching in the phormidolide, leptolyngbyalide and oocydin biosynthetic pathways, all of which use multiple ECH_2_ domains to install distinct β-branches (Fig. S19).^7,36,37^

Alternatively, in the context of module 11, BonI may be unable to function (pathway B, Fig. 6). This could arise from several possible mechanisms. First, the overall structural architecture of module 11 may impede access of BonI to the module 11 ACP_A_s and thereby prevent its enzymatic action. Second, BonI may have a strong preference for the short four carbon chain length polyketide it acts on at the beginning of the pathway and not recognise the longer, more complex polyketide present at module 11. Substrate control may also contribute to the in-*cis versus* in-*trans* control highlighted in the biosynthetic pathways above.

At this stage, it is unclear if a single pathway (kinetic control (A) or steric occlusion (B)) or a more complex interplay between both pathways (*i.e.*, the steric occlusion of BonI by the authentic polyketide substrate, coupled with in-*cis* kinetic control directed by the [KS]) is utilised to prevent decarboxylation of the carboxymethyl β-branch. Insights into this mechanism will have broad applicability for biosynthetic pathways that utilise duplicate ECH domains and enhance our knowledge of β-branching control mechanisms.

## 3 Conclusions

In summary, this study has identified a previously unannotated ACP_D_ within bongkrekic acid biosynthesis and successfully reconstituted the β-branching pathway with module 1 and module 11 ACP_A_s. ESMS assays confirmed that the newly identified ACP_D_, BonN, could undergo decarboxylation and subsequent aldol addition *via* BonG and an ACP_A_ to afford a HMG-ACP_A_ species. Divergent ECH_2_ processing of these HMG-ACP_A_s was interrogated to probe *endo*-β-branch (module 1) *versus* carboxymethyl β-branch (module 11) formation *via* the metabolically coupled ECH_1_/ECH_2_ pair, BonH/BonI. Our results confirm that the divergent processing at module 11 (on BonD_ACP3b) was not due to a lack of recognition between the ACP_A_ and BonI. Instead, a more complex mechanism must exist within the PKS to prevent BonI from acting at this stage.

To fully interrogate the mechanism for retention of the carboxymethyl β-branch, a series of more representative HMG-based polyketide substrates are required to assess whether BonI lacks specificity for late-stage polyketide intermediates. Since BonD does not contain a TE domain to release the polyketide chain, this suggests that an alternative chain release mechanism terminates PKS processing. This may require the unusual terminal [KS] domain, which could play a catalytic or non-catalytic role. Understanding the precise interplay of in-*cis* and in-*trans* components within the final module to prevent aberrant ECH_2_ processing by either discrete kinetic or steric control, or a combination of the two, is currently the subject of structural and biochemical investigation.

## Author contributions

Megan E. M. Hiseman: investigation, methodology, writing – original draft; Annabel P. Phillips: investigation, writing – original draft; Ciprian Chiriac: investigation; Liam J. Smith: writing – review & editing; John Crosby: funding acquisition, writing – review & editing; Christopher Williams: investigation; Christine L. Willis: funding acquisition, writing – review & editing, supervision; Ashley J. Winter: writing – original draft, investigation, methodology, supervision, conceptualisation; Matthew P. Crump: funding acquisition, project administration, supervision, conceptualisation, writing – review & editing.

## Conflicts of interest

There are no conflicts to declare.

## Supplementary Material

RA-015-D5RA05400A-s001

## Data Availability

The sequences for all Bon proteins can be found at accession JX173632 on NCBI. The sequence for the newly annotated BonN can be found at NCBI WP_013698117.1. Supplementary information: all experimental procedures. See DOI: https://doi.org/10.1039/d5ra05400a.
