# Supplementary material for: β-Branching in the biosynthesis of bongkrekic acid: a complex affair
Source: RSC Adv. 2025 Oct 28;15(48):40855–63. doi: 10.1039/d5ra05400a (PMC12560621; doi:10.1039/d5ra05400a)
Supplement: RA-015-D5RA05400A-s001 [file RA-015-D5RA05400A-s001.pdf]

Supplementary Information (SI) for RSC Advances.
This journal is © The Royal Society of Chemistry 2025

Electronic Supplementary information

β-Branching in the biosynthesis of bongkrekic acid: A complex affair

Megan E M Hiseman[a], Annabel P Phillips[a], Ciprian Chiriac[a], Liam Smith[a], John Crosby[a], Christopher Williams[a], Christine L Willis[a], Ashley J Winter*[a], Matthew P Crump*[a]

[a] School of Chemistry, University of Bristol, Bristol, BS8 1TS, UK.

*Corresponding authors: ash.winter@bristol.ac.uk; matt.crump@bristol.ac.uk

Methods

1. General

Reagents were purchased from Sigma-Aldrich, Thermo Fisher Scientific or Merck Life Science. Competent *E. coli* cells were purchased from Agilent (ArcticExpress (DE3) RIL), Thermo Fisher (One Shot BL21 Star (DE3)) and New England Biolabs (T7 Express and 5-α).

2. Plasmid generation

All nucleotide sequences were synthesised and subcloned into a pET151-D/TOPO vector with an N-terminal His_6-tag and a tobacco etch virus (TEV) cleavage site (ENLYFQ^G) by Thermo Fisher Scientific.

3. Expression and purification of proteins

Both MupN and CoaA/CoaD/CoaE were expressed and purified as described previously.[1, 2] Individual plasmids encoding the following (BonN, GbnF, BonF, BonA_ACP1a, BonA_ACP2a, BonD_ACP3b and BonH) were transformed into *E.coli* T7 Express cells. Cells were grown in LB media (37 °C) supplemented with carbenicillin (100 μg/mL) until OD_{600} = 0.6, then they were induced with 0.25-0.5 mM isopropyl β-D1-thiogalactopyranoside (IPTG) at 16 °C (16 h). Cell pellets were harvested by centrifugation (6000 rpm, 10 mins) and resuspended in buffer A (50 mM Tris-HCl, 500 mM NaCl, 10% (v/v) glycerol, pH 8.0) supplemented with 1 mM TCEP before storing at -20°C. BonG was transformed into One Shot BL21 Star (DE3) cells as described above, and cultures were supplemented with an additional 0.5 M sorbitol to promote solubility of BonG and were induced with 0.1 mM IPTG (16 °C, 16 h) before cell pellets were harvested by centrifugation and stored as described above.[3]

Plasmids encoding BonD_ACP1b and BonI were transformed into ArcticExpress RIL cells. Individual colonies were incubated in LB media supplemented with carbenicillin (100 μg/ml) and gentamycin (20 μg/ml) for 16 h at 37 °C. Overnight cultures were supplemented into fresh LB media (1:10 dilution) and grown to OD_{600} = 0.5-0.7 in LB media (30 °C) supplemented with carbenicillin (100 μg/ml), before induction with 0.25 mM IPTG at 10 °C for 24 h. Cell pellets were harvested and stored as described above.

Resuspended cell pellets were sonicated and the soluble fraction was purified by immobilised metal affinity chromatography (IMAC) through a HiTrap 5 mL HP Ni column (GE Healthcare). Proteins were eluted using a linear gradient of 6-100% Buffer B (50 mM Tris-HCl, 500 mM NaCl, 10% (v/v) glycerol, 800 mM imidazole, pH 8.0). Samples were further purified by size exclusion chromatography (SEC) via either a HiPrep 26/60 Sephacryl S100 or S200 column (GE Healthcare) in Buffer C (25 mM Tris-HCl, 150 mM NaCl, pH 7.5, 1 mM DTT) or Buffer D (25 mM Tris-HCl, 500 mM NaCl, pH 7.5, 1 mM DTT) respectively. Proteins were then concentrated and either immediately used or stored at -20 °C. Purified protein samples (50 μM) were analysed by analytical SEC with either a Superdex 75 10/300 or Superdex 200 increase 10/300 GL column (GE Healthcare) calibrated using molecular weight standards (GE Healthcare).[4]

4. ESMS Assays

Assay samples were desalted prior to ESMS analysis using a C_4 ZipTip (Merck) according to the manufacturer's instructions. Denatured samples were analysed on a Synapt G2-Si(Waters) fitted with a TriVersa NanoMate (Advion) using the following parameters: capillary voltage 1.5 kV; sample cone 10 V; trap collision energy 10 V. Using a positive mode source, spectra were acquired over 200-3000 m/z and analysed using MassLynx 4.1. For Ppant ejection assays the MS/MS functionality was used to isolate an appropriate charge state. The transfer collision energy was increased to induce fragmentation (typically 5 – 20 V) and spectra were collected from 200 – 1000 m/z.

4.1. BonF ESMS Assay

100 µM ACP (BonN, GbnF or BonD_ACP3b, BonA_ACP1a or BonD_ACP1b) was upgraded with malonyl-CoA (100 µl reactions) as previously described and desalted via a Zeba column (Thermo Fisher) equilibrated with Buffer A.[2] Malonylated ACPs (50 µM) were then incubated at room temperature for 3 h with 5 µM BonF in Buffer A. Interrogation of the acylated intermediate by ESMS and Ppant ejection of ACP species were used to determine the outcome of the reaction.

4.2 BonG ESMS Assay

100 µM ACP (BonN, GbnF, BonD_ACP3b, BonA_ACP1a or BonD_ACP1b; 50 µM BonA_ACP2a) was upgraded with propionyl-CoA, acetyl-pantetheine or acetoacetyl-pantetheine, the latter two were synthesized as previously described[1, 5] ACPs were desalted into Buffer A with a Zeba column (Thermo Fisher). Derivatised ACP_D and ACP_A candidates (50 µM each)were incubated at room temperature with 5 µM BonG for 3 h. Due to low yields only 25 µM Acac-BonA_ACP2a could be utilised in assays as an ACP_A candidate. Reactions were monitored by ESMS and Ppant ejection analysis.

4.3 ECH ESMS Assay

100 µM ACP (BonD_ACP3b, BonA_ACP1a and BonD_ACP1b) or 50 µM ACP (BonA_ACP2a) was upgraded with HMG-CoA and desalted through a Zeba column (Thermo Fisher) equilibrated with Buffer D. HMG-ACPs (50 µM) were incubated with BonH and BonI (5 µM each) in Buffer D at room temperature for 1-3 h. Reactions were monitored by ESMS and Ppant ejection analysis after 1hr.

5. ^{1}H NMR

All protein NMR experiments were performed on a Bruker Avance III HD 700 MHz spectrometer (Bruker, Billerica, MA, USA) equipped with a 1.7 mm triple-resonance micro-cryoprobe using standard pulse sequences from the Bruker pulse library. One-dimensional ^{1}H-NMR spectra were acquired for ACP samples (100 µM) in Buffer C. Samples were acquired at 298 K in 10% D_20 and referenced using 4,4-dimethyl-4-silapentane-1-sulfonic acid (DSS).

6. Circular Dichroism

Spectra for ACP samples of BonN and GbnF (both 20 µM in buffer C) were recorded at 20 °C on a JASCO J-1500 spectrophotometer and averaged over 8 scans (200-270 nm range). The Spectra Manager™ suite was used for background subtraction and smoothing.

7. Phylogenetic tree analysis

Amino acid sequences from 51 characterised *trans*-acting ACP_D sequences, were manually extracted from *cis* and *trans*-AT PKS clusters, alongside the candidate WP_013698117.1 sequence and the 14 modular cis-ACPs from the BonA-BonD PKS. The 7 modular *cis*-ACPs encoded within the Erythromycin BGC were chosen as outgroup ACP domains (EryAI_ACP1-3, EryAII_ACP1-2 and EryAIII_ACP1-2) to root the phylogenetic tree. The sequences were aligned using the MUSCLE algorithm[6] with default settings and a Maximum-likelihood phylogenetic tree was computed using Mega-X[7] with 500 bootstrap iterations using the LG+G+I substitution model, with all gap sites included. The bootstrapped tree was visualised using FigTree.

Burkholderia gladioli pv *cocovenenas* (taxid: 28095)			
Accession number	Query Cover	Amino acids	Sequence identity
WP_036053932.1	100%	82	100.00%
WP_186184515.1	100%	82	98.78%
WP_186060070.1	100%	82	98.78%
WP_186229213.1	100%	82	98.78%
**WP_013698117.1**	**96%**	**85**	**59.49%**
WP_186070908.1	96%	85	58.23%
WP_017918653.1	96%	85	58.23%
WP_013689030.1	96%	79	54.43%
Burkholderia gladioli BSR3 (taxid: 999541)			
**WP_013698117.1**	**96%**	**85**	**59.49%**
WP_013689030.1	96%	79	54.43%

Table S1. ACP hits for Protein BLAST searches using the amino acid sequence of GbnF (NCBI accession: WP_036053932.1) as a probe. Hits with greater than 50% sequence identity are shown. WP_013698117.1 (bold) was conserved in both strains.

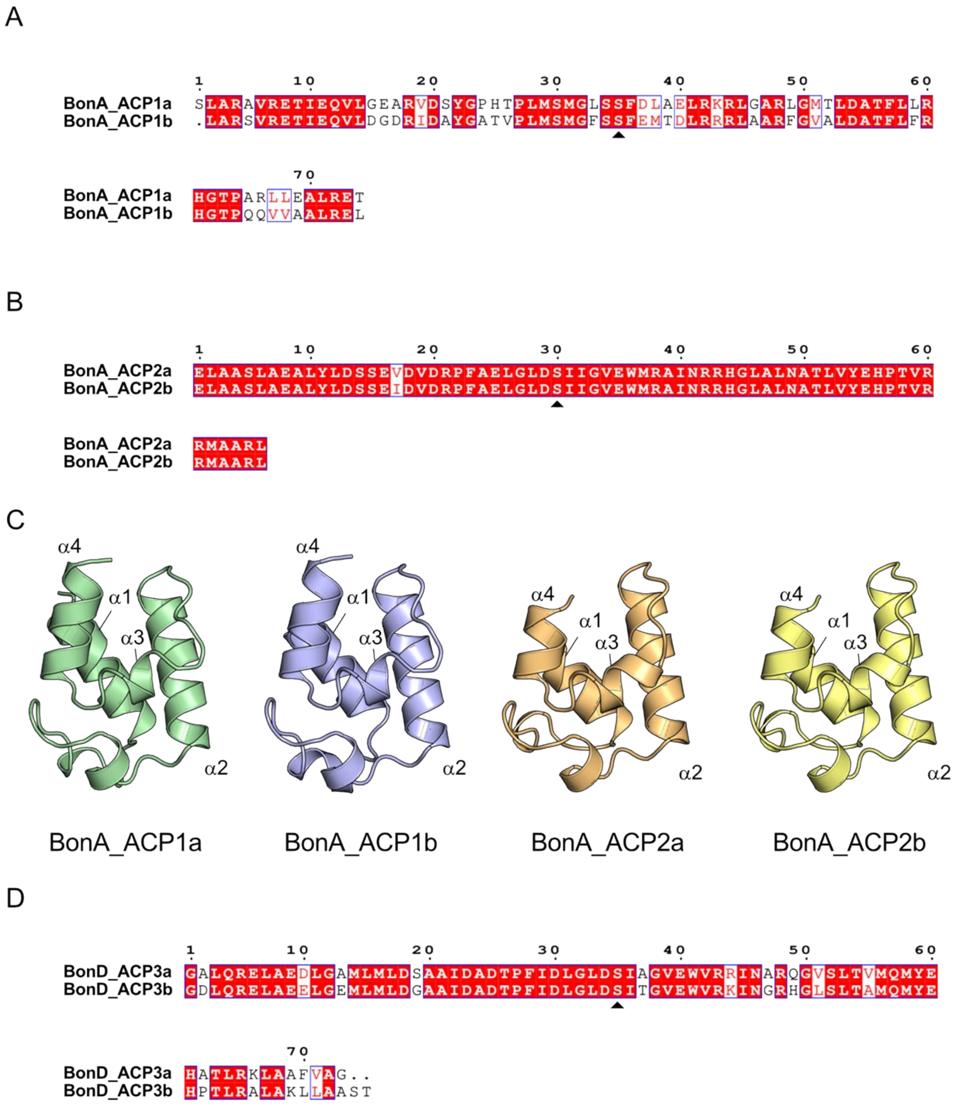

Figure S1. Identification of tandem ACP domains in the load module and module 1 of the type I PKS BonA. A) Sequence alignment of BonA_ACP1a (previously BonA_ACP1) with the tandem partner BonA_ACP1b (63.51% sequence identity).[8] B) Sequence alignment of BonA_ACP2a (previously BonA_ACP2) with the tandem partner BonA_ACP2b (98% sequence identity). C) AlphaFold models of each putative ACP indicating the structural arrangement of a four-helix bundle for each sequence.[9] D) Sequence alignment of BonD_ACP3a and BonD_ACP3b (76% sequence identity). For each ACP alignment the black arrow refers to the conserved serine phosphopantetheine attachment site at the base of helix 2.

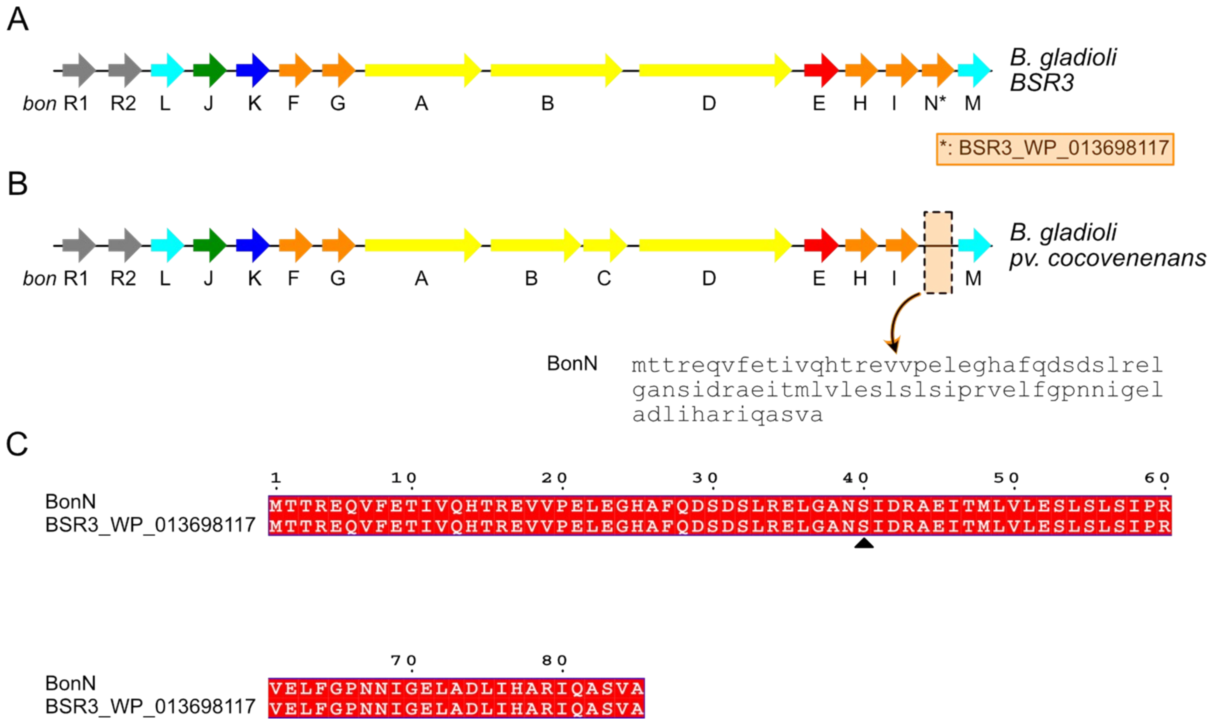

Figure S2. Reanalysis of the *B. gladioli* BSR3 (A) and *B. gladioli* pv. *cocovenenans* (B) *bon* BGC to identify the unannotated putative donor ACP domain (Referred to as BSR3_WP_013698117 for *B. gladioli* BSR3 and BonN for *B. gladioli* pv. *cocovenenans* . A black arrow refers to the conserved serine phosphopantetheine attachment site of the ACP (blue). (C) Sequence alignment of BonN and BSR3_WP_013698117.

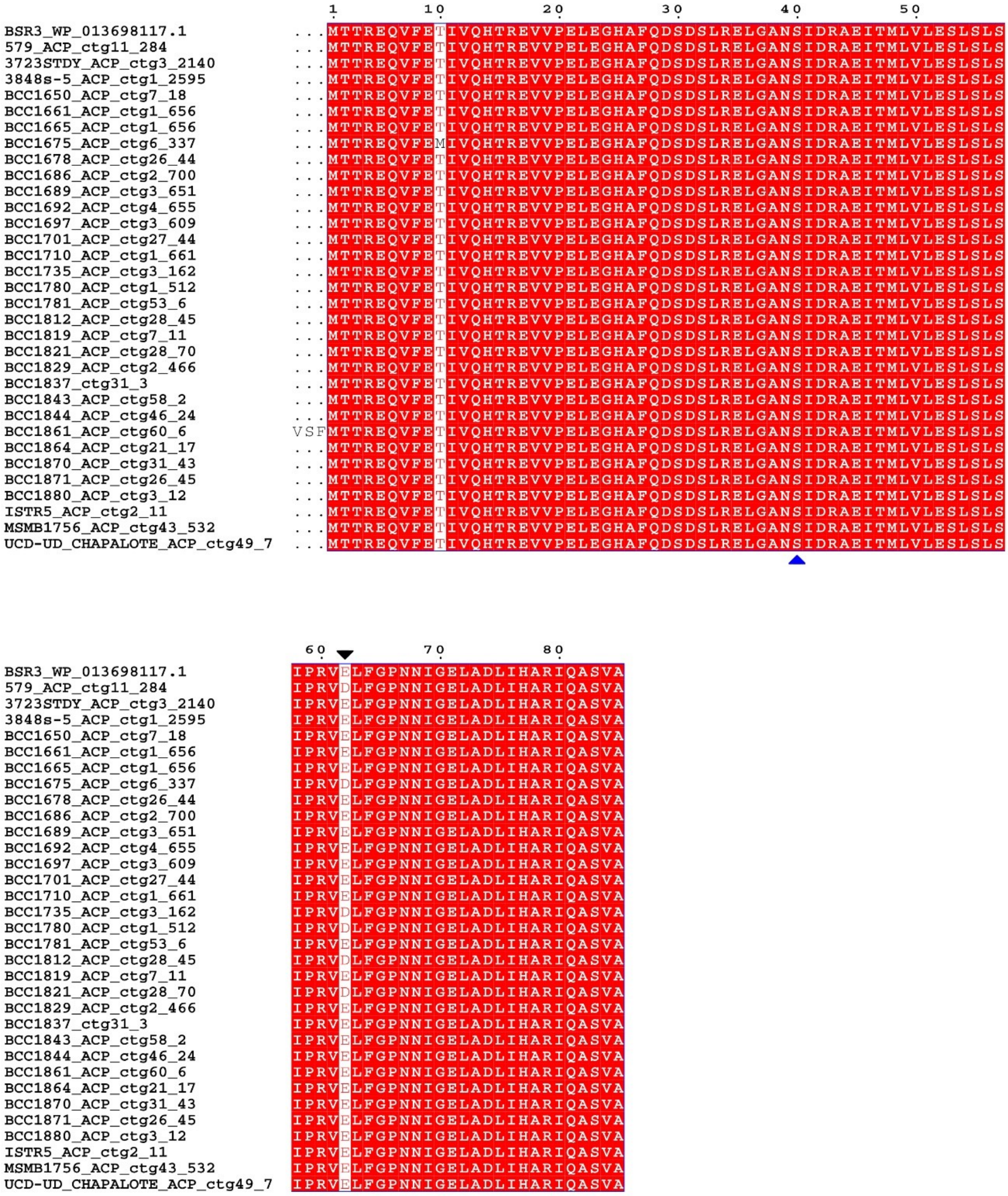

Figure S3. Sequences of all putative donor ACP sequences found in 33 *B. gladioli* strains that contain a *bon* BGC. The blue arrow refers to the conserved serine phosphopantetheine attachment site, while the black arrow marks a single nucleotide polymorphism site across all ACP sequences.

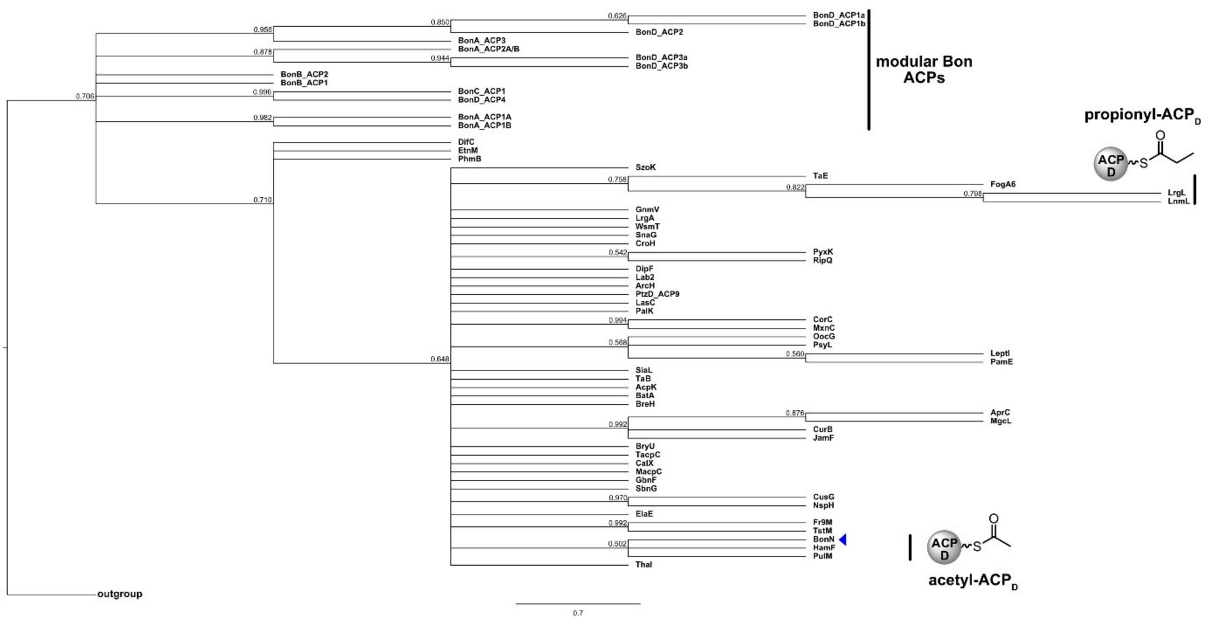

Figure S4. The maximum-likelihood phylogenetic tree of 51 ACP_Ds, including BonN, and 14 modular Bon ACPs. Seven modular erythromycin ACPs from EryAI-AIII PKSs were used as the outgroup. The blue arrow marks BonN.

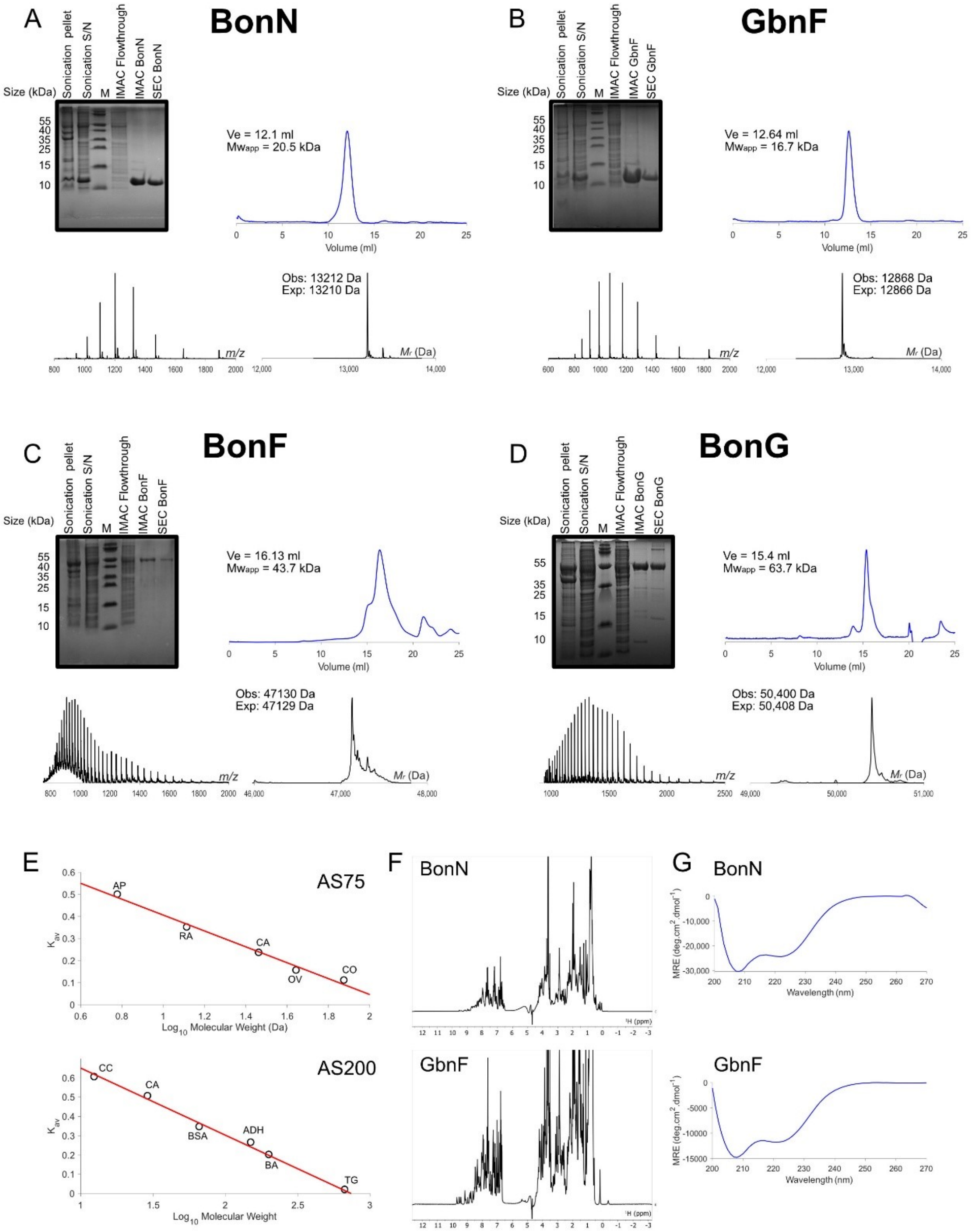

Figure S5. Protein purification and characterisation. A) **BonN**. SDS-PAGE following the purification after sonication, Ni^{2+} immobilised metal affinity chromatography (IMAC) and size exclusion chromatography (SEC). Analytical SEC (AS75) showing His_6-BonN eluting as a monomer and non-native ESMS confirming the correct sequence of BonN. B) **GbnF**. As A). SDS-PAGE following the purification after sonication, IMAC and SEC, with analytical SEC (AS75) showing GbnF eluting as a monomer and non-native ESMS confirming the correct sequence. C) **BonF**. As A). SDS-PAGE following the purification after sonication, IMAC and SEC. Analytical SEC (AS200) showing BonF eluting as a monomer and non-native ESMS confirming the correct sequence. D) **BonG**. As A). SDS-PAGE following the purification after sonication, IMAC and SEC. Analytical SEC (AS200) showing BonG eluting as a monomer and non-native ESMS confirming the correct sequence. E) Calibration curve for the analytical S75 and analytical S200 column using the following calibrants: aprotinin (AP), ribonuclease A (RA), carbonic anhydrase (CA), ovalbumin (OV), conalbumin (CO), cytochrome C (CC), bovine serum albumin (BSA), alcohol dehydrogenase (ADH), β-amylase (BA) and thyroglobulin (TG). F) ^{1}H NMR spectra of BonN and GbnF indicating folded protein species. G) CD spectra of BonN and GbnF, both displaying an α-helical profile.

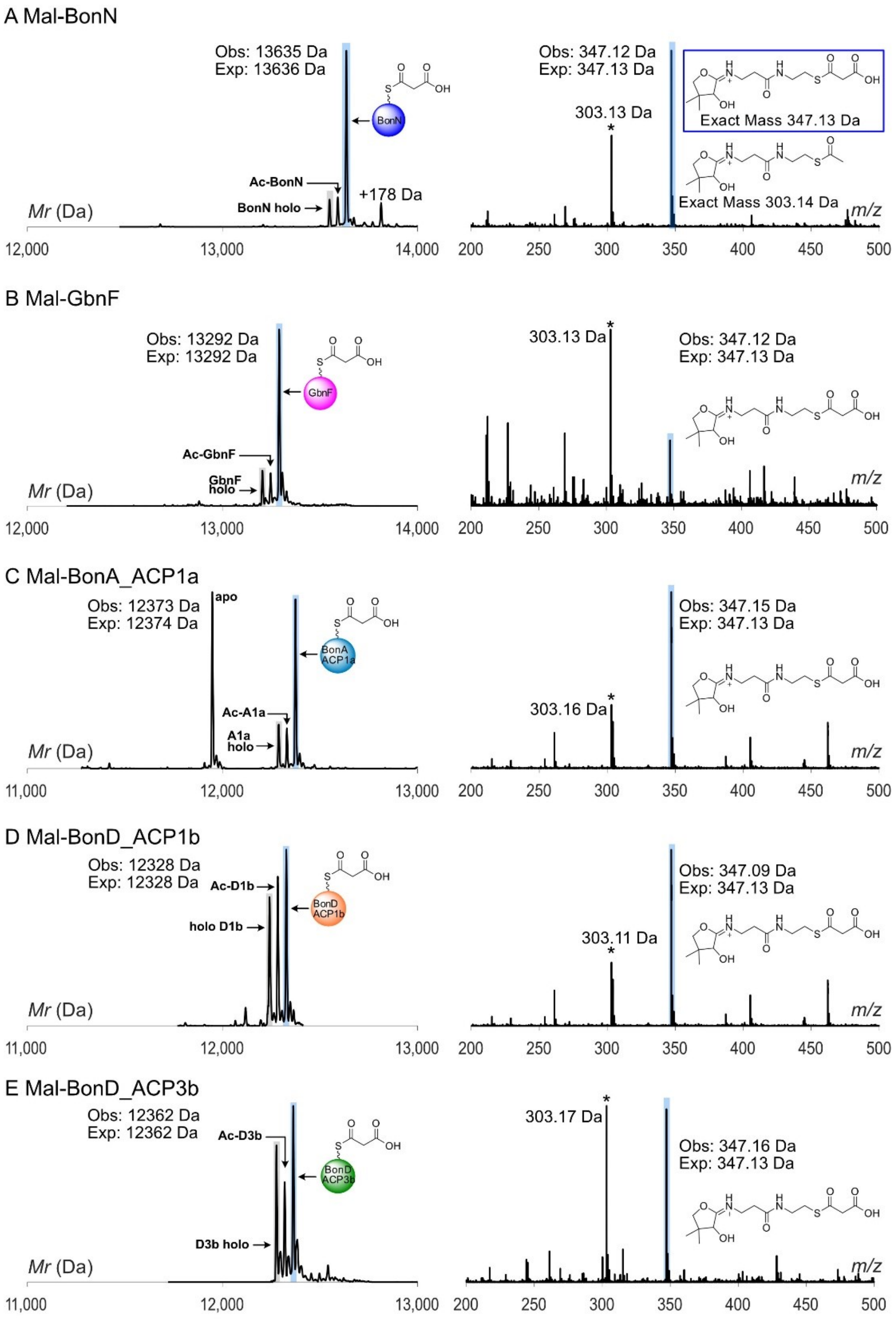

Figure S6. Malonyl (Mal) upgrades of Bon ACPs and GbnF. A) Deconvoluted spectrum of Mal-BonN (obs: 13,635 Da, exp: 13,636 Da) and the Ppant ejection for this species (obs: 347.12 Da, exp: 347.13 Da), an acetyl Ppant ion adduct (exp: 303.14 Da) is also observed (*). +178 refers to phosphogluconoylation of the His_6 tag (+178 Da). B) Deconvoluted spectrum of Mal-GbnF (obs: 13,292 Da, exp: 13,292 Da) and the Ppant ejection for this species (obs: 347.12 Da, exp: 347.13 Da). C) Deconvoluted spectrum of Mal-BonA_ACP1a (obs: 12,370 Da, exp: 12,374 Da) and the Ppant ejection for this species (obs: 347.15 Da, exp: 347.13 Da), D) Deconvoluted spectrum of Mal-BonD_ACP1b (obs: 12,328 Da, exp: 12,328 Da) and the Ppant ejection for this species (obs: 347.09 Da, exp: 347.13 Da), E) Deconvoluted spectrum of Mal-BonD_ACP3b (obs: 12,362 Da, exp: 12,362 Da) and the Ppant ejection for this species (obs: 347.16 Da, exp: 347.13 Da).

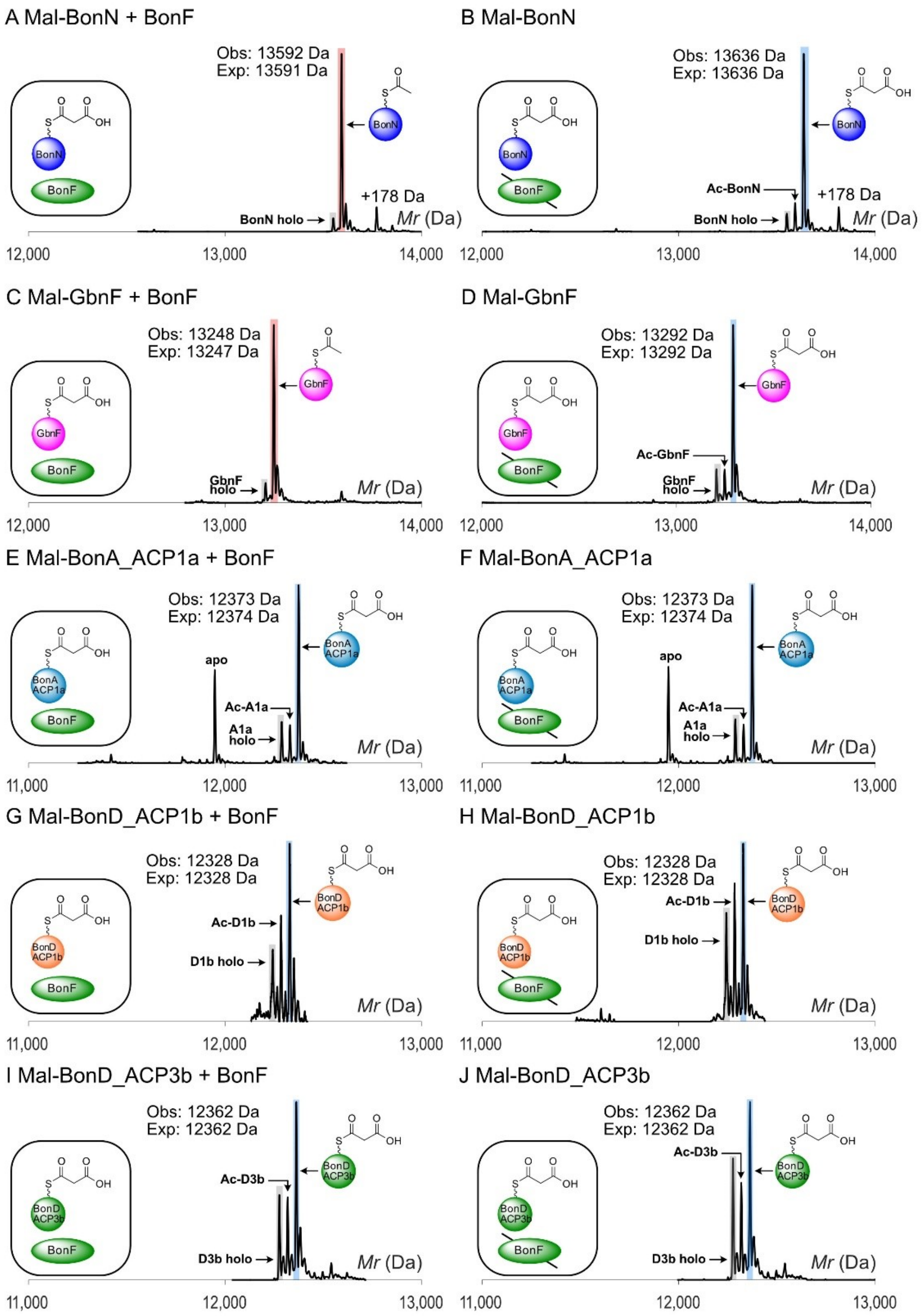

Figure S7. Decarboxylation assays utilising BonF. A) Deconvoluted spectrum of malonyl (Mal)-BonN when incubated with BonF (left) (obs: 13,592 Da, exp: 13,591 Da). B) Incubation with denatured BonF (right) (obs: 13,636 Da, exp: 13,636 Da). C) Deconvoluted spectrum of Mal-GbnF when incubated with BonF (left) (obs: 13,248 Da, exp: 13,247 Da). D) Incubation with denatured BonF (right) (obs: 13,292 Da, exp: 13,292 Da). E) Deconvoluted spectrum of Mal-BonA_ACP1a when incubated with BonF (left) (obs: 12,373 Da, exp: 12,374 Da). F) Incubation with denatured BonF (right) (obs: 12,373 Da, exp: 12,374 Da). G) Deconvoluted spectrum of malonyl-BonD_ACP1b when incubated with BonF (left) (obs: 12,328 Da, exp: 12,328 Da). H) Incubation with denatured BonF (right) (obs: 12,328 Da, exp: 12,328 Da). I) Deconvoluted spectrum of Mal-BonD_ACP3b when incubated with BonF (left) (obs: 12,362 Da, exp: 12,362 Da). J) Incubation with denatured BonF (right) (obs: 12,362 Da, exp: 12,362 Da).

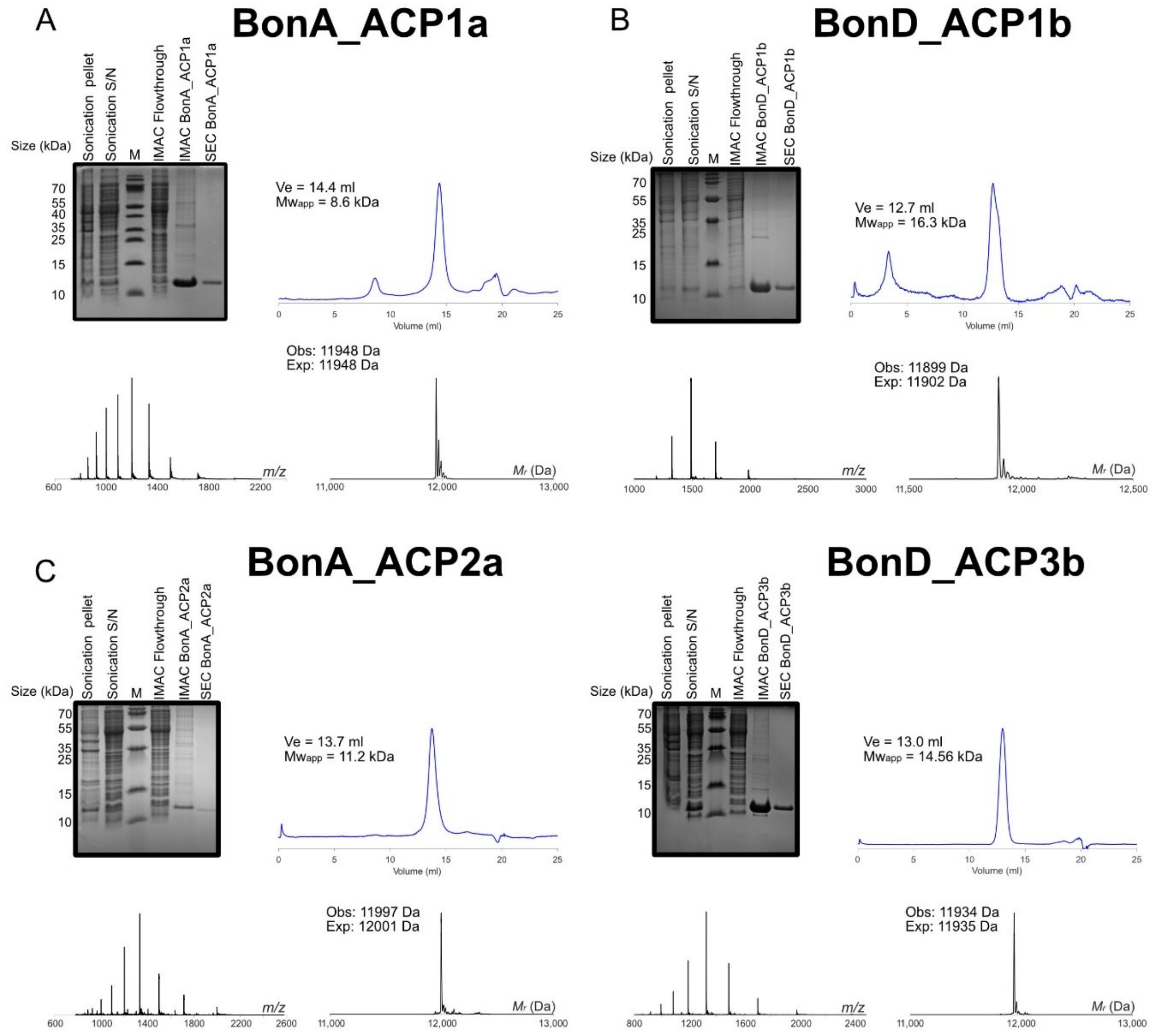

Figure S8. Protein purification and characterisation. A) **BonA_ACP1a**. SDS-PAGE following the purification after sonication, IMAC and SEC. Analytical SEC (AS75) showing His_6-BonA_ACP1a eluting as a monomer and non-native ESMS confirming the correct sequence of BonA_ACP1a. B) **BonD_ACP1b**. As A) SDS-PAGE following the purification after sonication, IMAC and SEC, with analytical SEC (AS75) showing elution of BonD_ACP1b as a monomeric species and non-native ESMS confirming the correct sequence. C) **BonA_ACP2a**. As A). SDS-PAGE following the purification after sonication, IMAC and SEC. Analytical SEC (AS75) showing BonA_ACP2a eluting as a monomer and non-native ESMS confirming the correct sequence. D) **BonD_ACP3b**. As A). SDS-PAGE following the purification after sonication, IMAC and SEC. Analytical SEC (AS75) showing BonD_ACP3b eluting as a monomer and non-native ESMS confirming the correct sequence.

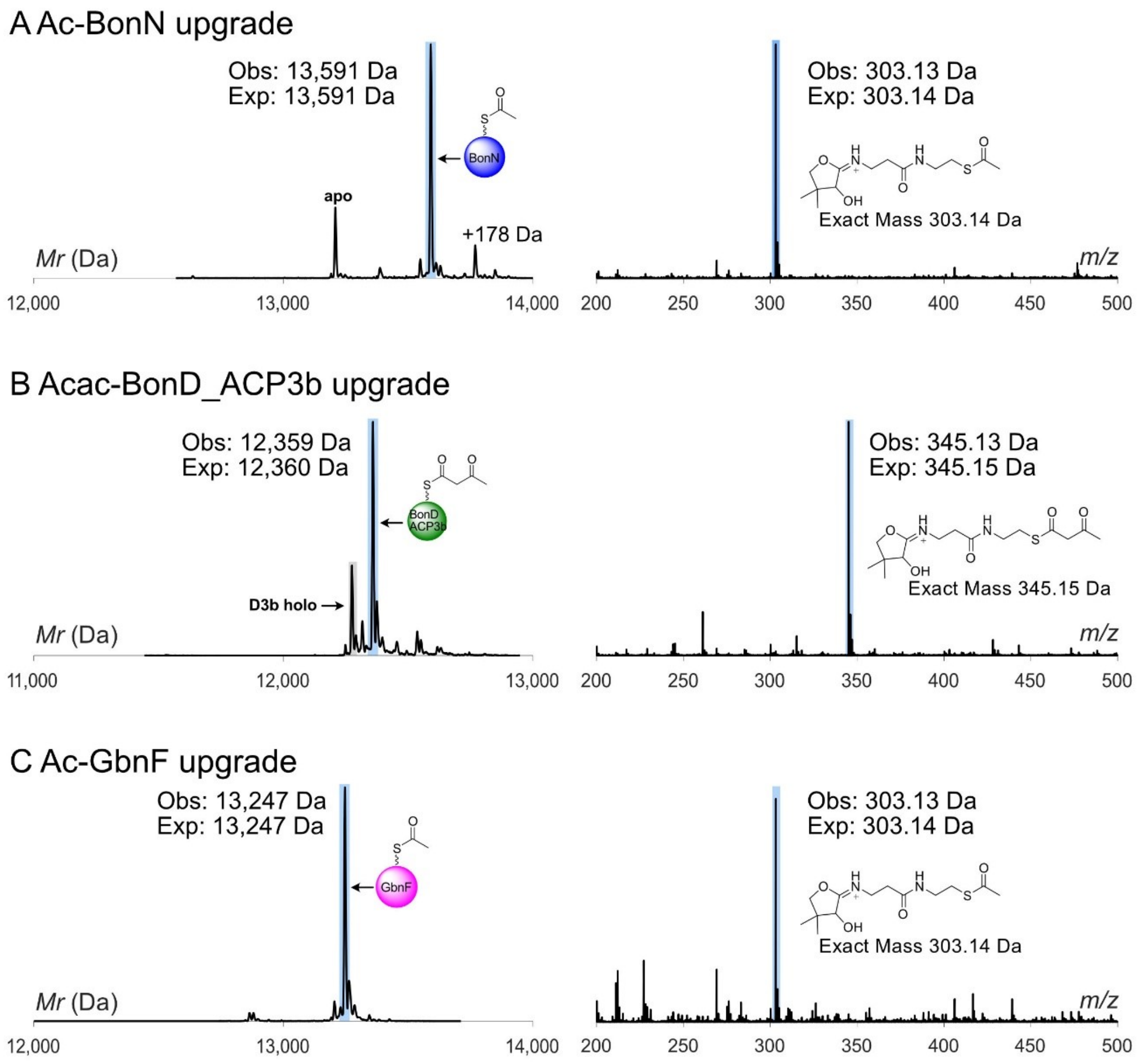

Figure S9. Upgrades of Bon ACPs and GbnF. A) Deconvoluted spectrum of acetyl (Ac)-BonN (obs: 13,591 Da, exp: 13,591 Da) and the Ppant ejection for this species (obs: 303.13 Da, exp: 303.14 Da). B) Deconvoluted spectrum of acetoacetyl (Acac)-BonD_ACP3b (obs: 12,359 Da, exp: 12,360 Da) and the Ppant ejection for this species (obs: 345.13 Da, exp: 345.15 Da). C) Deconvoluted spectrum of acetyl-GbnF (obs: 13,247 Da, exp: 13,247 Da) and the Ppant ejection for this species (obs: 303.13 Da, exp: 303.14 Da).

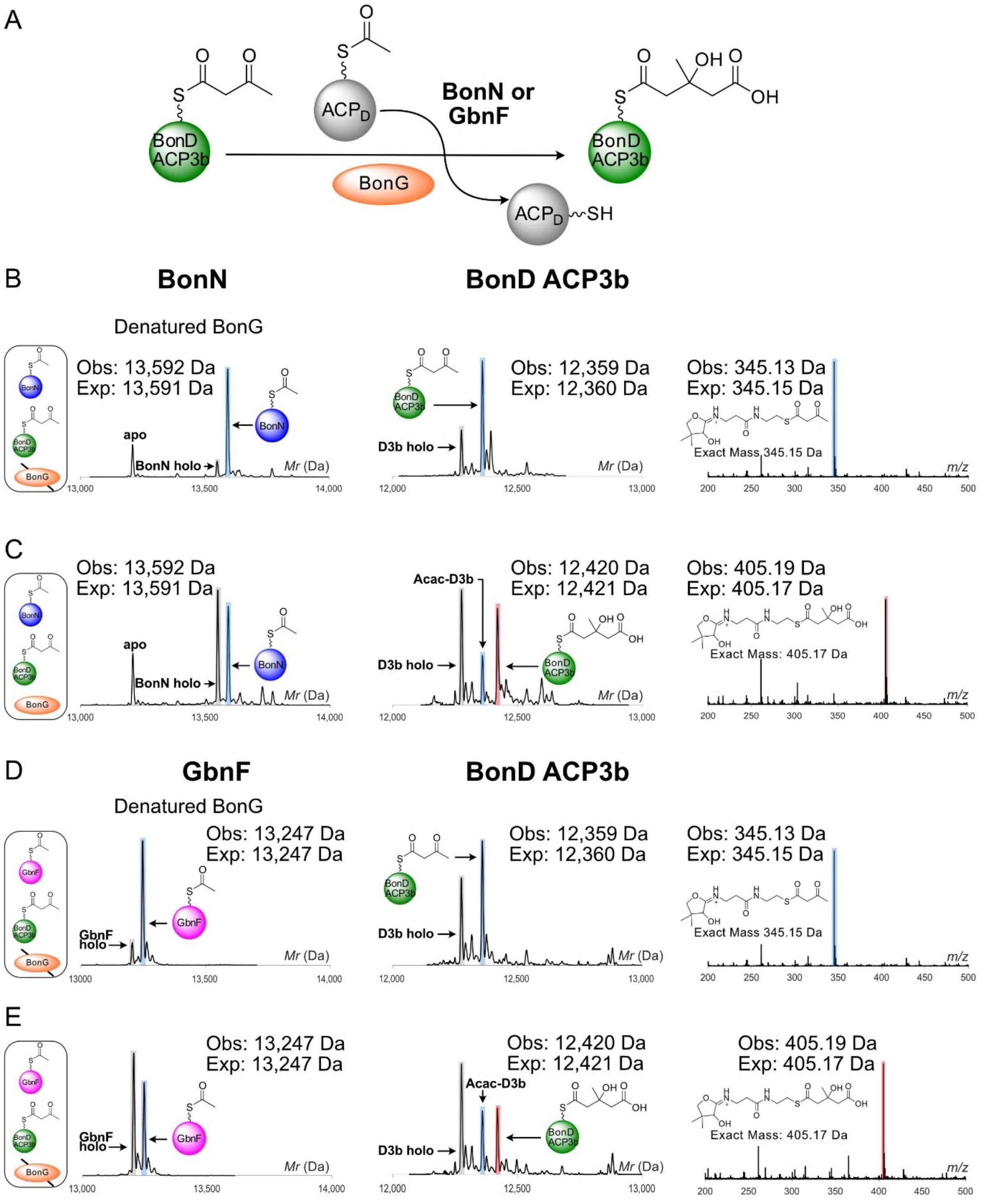

Figure S10. HMGS assays utilising BonG. A) Proposed scheme for aldol addition catalysed by BonG. B) Deconvoluted spectra of BonN (left) (obs: 13,592 Da), exp: 13,591 Da) and BonD_ACP3b (middle) (obs: 12,359 Da, exp: 12,360 Da) when incubated with denatured BonG. Ppant ejection (right) shows the ejection ion for acetoacetyl (Acac)-BonD_ACP3b (obs: 345.13 Da, exp: 345.15 Da). C) Deconvoluted spectra of BonN (left) (obs: 13,592 Da), exp: 13,591 Da) and BonD_ACP3b (middle) (obs: 12,359 Da, exp: 12,360 Da) when incubated with BonG. Ppant ejection (right) shows the ejection ion for HMG-BonD_ACP3b (obs: 405.19 Da, exp: 405.17 Da). D) Deconvoluted spectrum of GbnF (left) (obs: 13,247 Da, exp: 13,247 Da) and BonD_ACP3b (middle) (obs: 12,359 Da, exp: 12,360 Da) when incubated with denatured BonG. Ppant ejection (right) shows the ejection ion for acetoacetyl-BonD_ACP3b (obs: 345.13 Da, exp: 345.15 Da). E) Deconvoluted spectrum of GbnF (left) (obs: 13,247 Da, exp: 13,247 Da) and BonD_ACP3b (middle) (obs: 12,420 Da, exp: 12,421 Da) when incubated with BonG. Ppant ejection (right) shows the ejection ion for HMG-BonD_ACP3b (obs: 405.19 Da, exp: 405.17 Da).

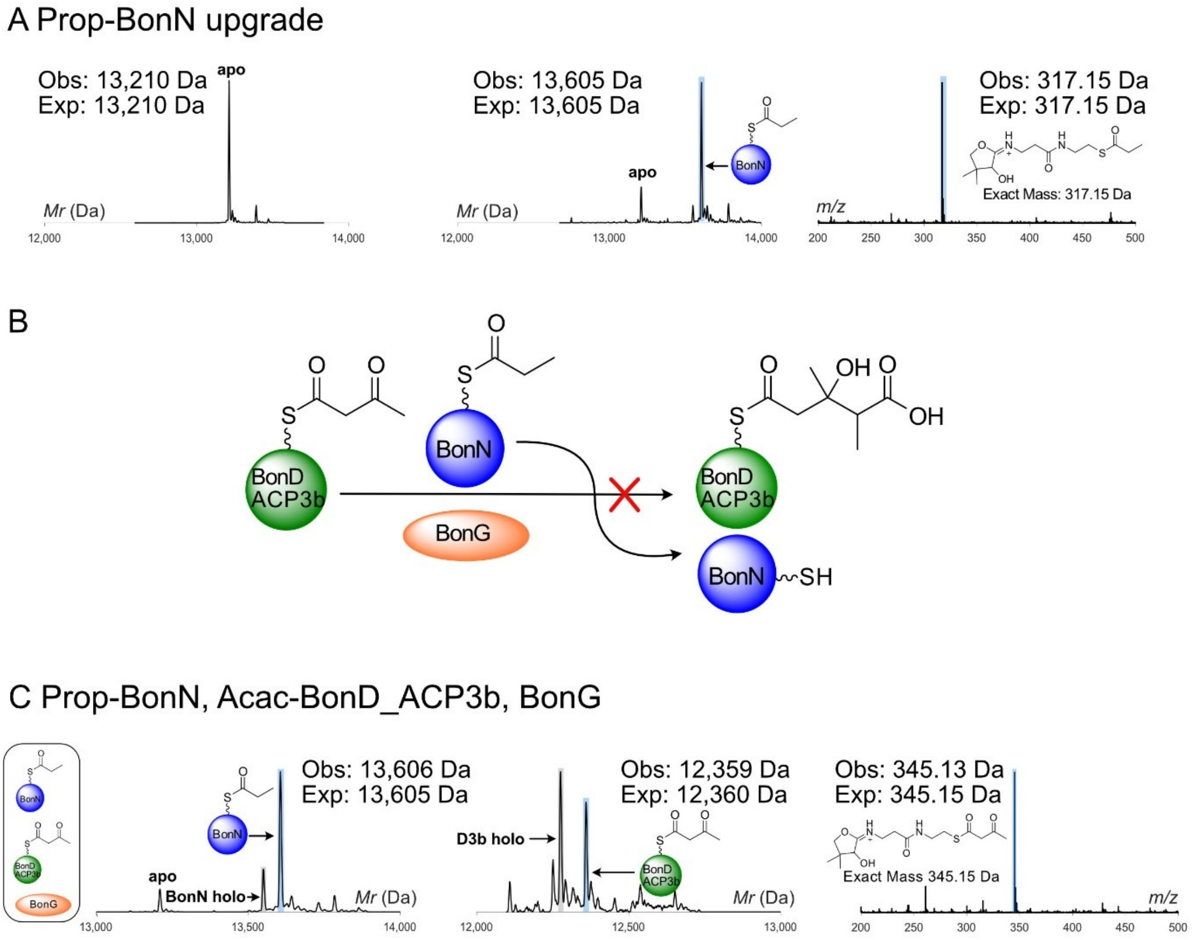

Figure S11. HMGS assay utilising propionyl-BonN. A) Deconvoluted spectrum of propionyl (Prop)-BonN (obs: 13,605 Da, exp: 13,605 Da) and the Ppant ejection for this species (obs: 317.15 Da, exp: 317.15 Da), B) Proposed scheme for aldol addition catalysed by BonG utilising propionyl (Prop)-BonN and acetoacetyl (Acac)-BonD_ACP3b. C) Deconvoluted spectra of Prop-BonN (left) (obs: 13,606 Da, exp: 13,605 Da) and Acac-BonD_ACP3b (middle) (obs: 12,359 Da, exp: 12,360 Da) when incubated with BonG. Ppant ejection (right) shows the ejection ion for Acac-BonD_ACP3b (obs: 345.13 Da, exp: 345.15 Da).

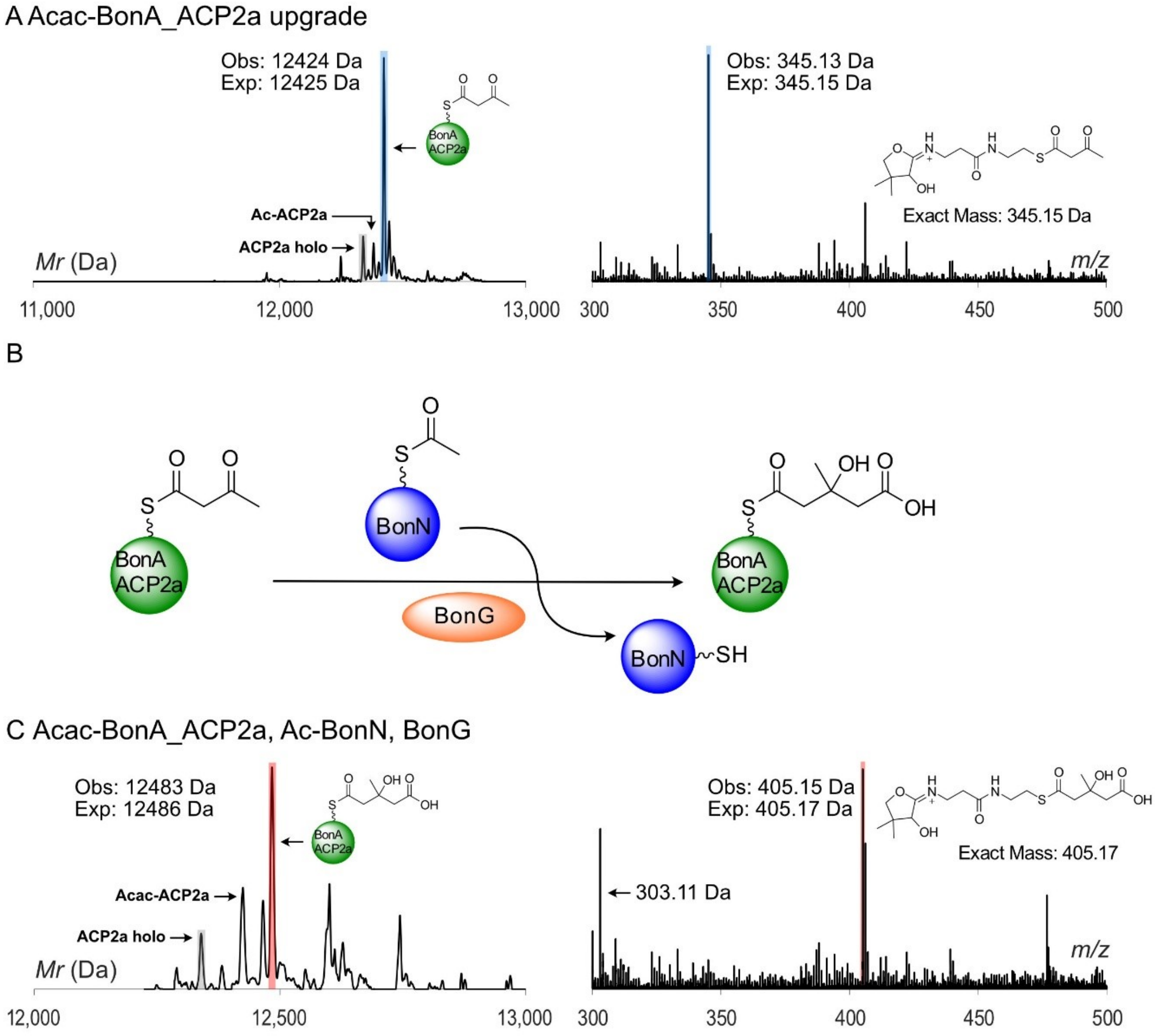

Figure S12. BonG HMGS assays utilising acetoacetyl (Acac)-BonA_ACP2a. A) Deconvoluted spectrum of Acac-BonA_ACP2a (obs: 12,424 Da, exp: 12,425 Da) and the Ppant ejection for this species (obs: 345.13 Da, exp: 345.15 Da). B) Proposed scheme for aldol addition catalysed by BonG utilising Acac-BonA_ACP2a. C) Deconvoluted spectrum of BonA_ACP2a (left) when incubated with BonG and acetyl-BonN to form HMG-BonA_ACP2a (obs: 12,483 Da, exp: 12,486 Da). Ppant ejection (right) shows the ejection ion for HMG-BonA_ACP2a (obs: 405.15 Da, exp: 405.17 Da).

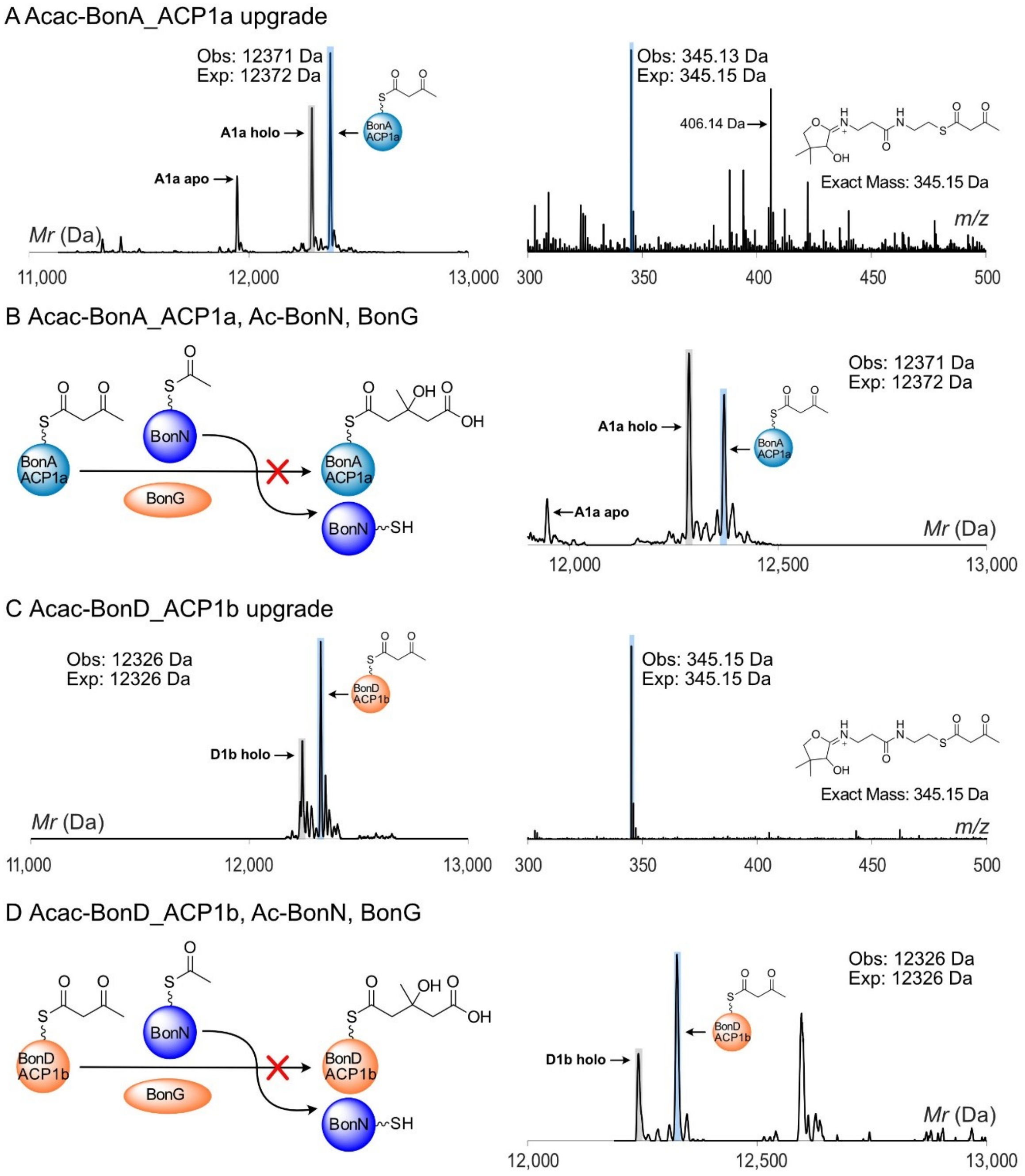

Figure S13. BonG HMGS control assays utilising acetoacetyl (Acac)-BonA_ACP1a and Acac_BonD_ACP1b. A) Deconvoluted spectrum of Acac-BonA_ACP1a (obs: 12,371 Da, exp: 12,372 Da) and the Ppant ejection for this species (obs: 345.13 Da, exp: 345.15 Da). B) Proposed scheme for aldol addition catalysed by BonG utilising Acac-BonA_ACP1a (left) and deconvoluted spectrum of Acac-BonA_ACP1a when incubated with acetyl (Ac)-BonN and BonG (obs: 12,371 Da, exp: 12,372 Da). C) Deconvoluted spectrum of Acac-BonD_ACP1b (obs: 12,326 Da, exp: 12,326 Da) and the Ppant ejection for this species (obs: 345.15 Da, exp: 345.15 Da). D) Proposed scheme for aldol addition catalysed by BonG utilising Acac-BonD_ACP1b (left) and deconvoluted spectrum of Acac-BonD_ACP1b when incubated with Ac-BonN and BonG (obs:12,326 Da, exp:12,326 Da). Both Acac-BonA_ACP1a and Acac-BonD_ACP1b did not turnover.

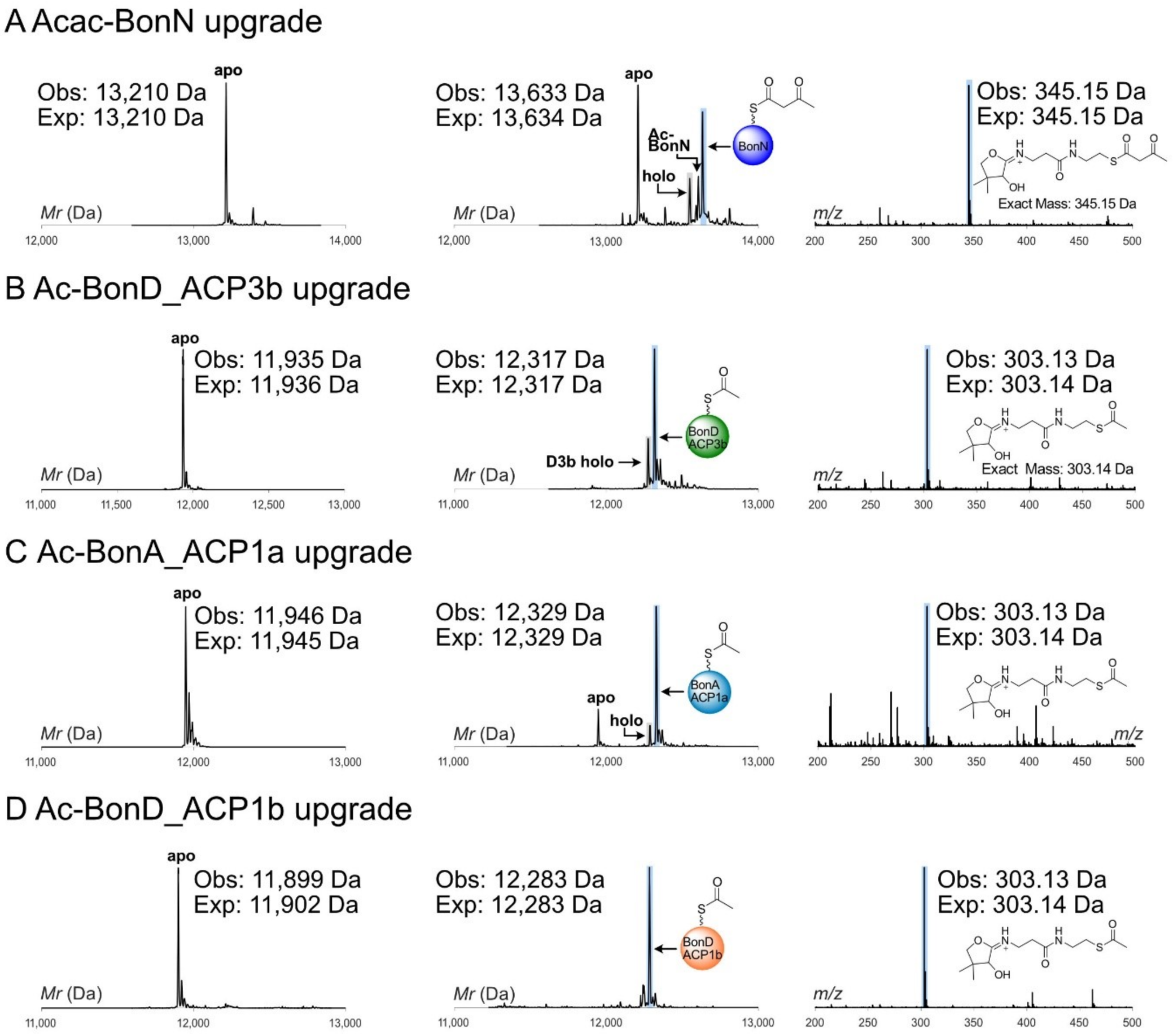

Figure S14. Upgrades of Bon ACPs. A) Deconvoluted spectrum of acetoacetyl (Acac)-BonN (obs: 13,633 Da, exp: 13,634 Da) and the Ppant ejection for this species (obs: 345.15 Da, exp: 345.15 Da). B) Deconvoluted spectrum of acetyl (Ac)-BonD_ACP3b (obs: 12,317 Da, exp: 12,317 Da) and the Ppant ejection for this species (obs: 303.13 Da, exp: 303.14 Da). C) Deconvoluted spectrum of Ac-BonA_ACP1a (obs: 12,329 Da, exp: 12,329 Da) and the Ppant ejection for this species (obs: 303.13 Da, exp: 303.14 Da). D) Deconvoluted spectrum of Ac-BonD_ACP1b (obs: 12,283 Da, exp: 12,283 Da) and the Ppant ejection for this species (obs: 303.13 Da, exp: 303.14 Da).

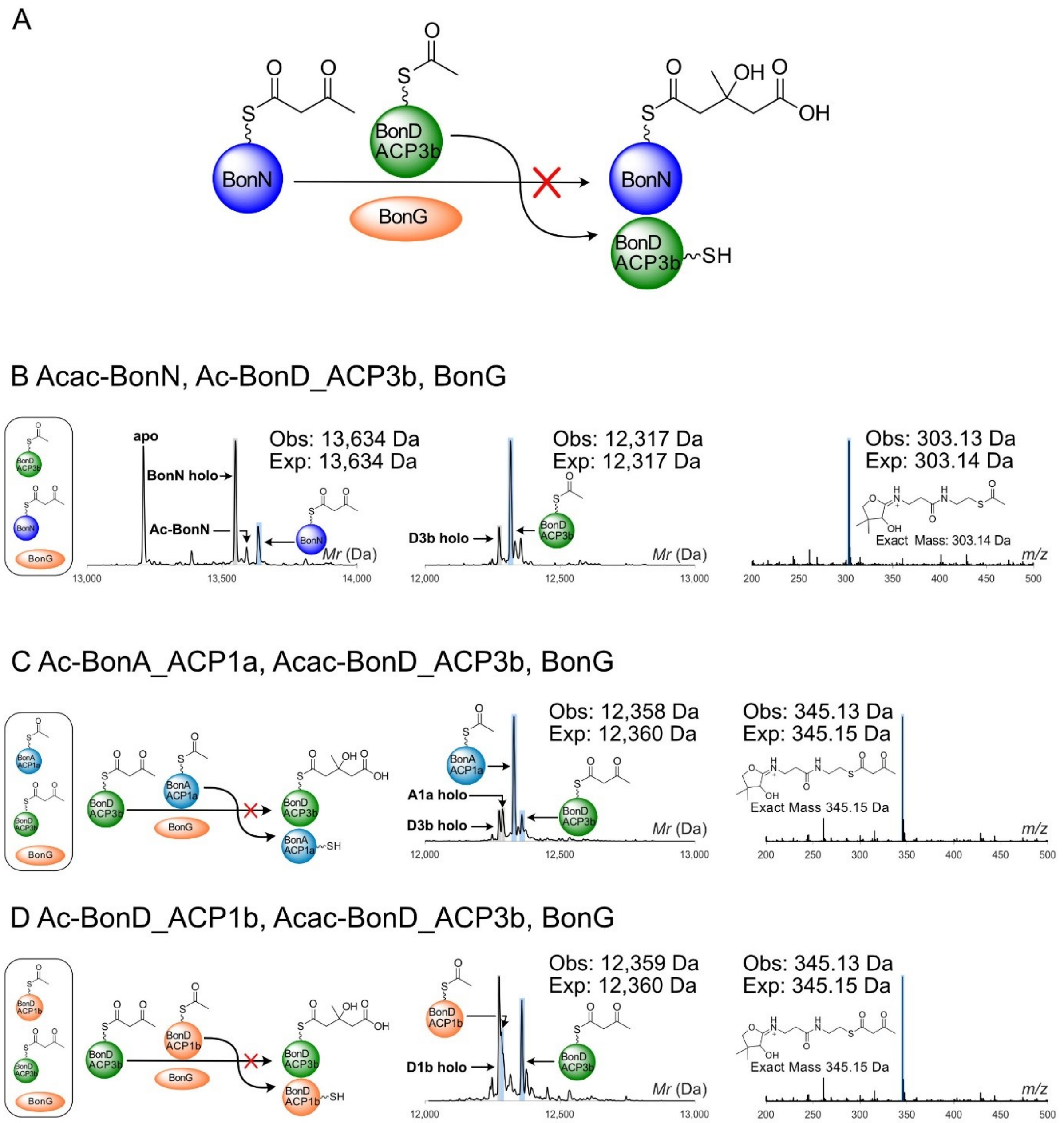

Figure S15. Control BonG HMGS assays. A) Proposed scheme for aldol addition catalysed by BonG utilising Acac-BonN and acetyl (Ac)-BonD_ACP3b. B) Deconvoluted spectra of Acac-BonN (left) (obs: 13,634 Da, exp: 13,634 Da) and Ac-BonD_ACP3b (middle) (obs: 12,317 Da, exp: 12,317 Da) when incubated with BonG. Ppant ejection (right) shows the ejection ion for Ac-BonD_ACP3b (obs: 303.13 Da, exp: 303.14 Da). C) Proposed HMGS scheme using Ac-BonA_ACP1a (left). Deconvoluted spectrum of Ac-BonA_ACP1a and Acac-BonD_ACP3b (obs: 13,358 Da, exp: 13,360 Da) (middle) when incubated with BonG. Ppant ejection (right) shows the ejection ion for Acac-BonD_ACP3b (obs: 345.13 Da, exp: 345.15 Da). D) Proposed HMGS scheme using Ac-BonD-ACP1b (left). Deconvoluted spectrum of Ac-BonD_ACP1b and Acac-BonD_ACP3b (obs: 12,359 Da, exp: 12,360 Da) (middle) when incubated with BonG. Ppant ejection (right) shows the ejection ion for Acac-BonD_ACP3b (obs: 345.13 Da, exp: 345.15 Da).

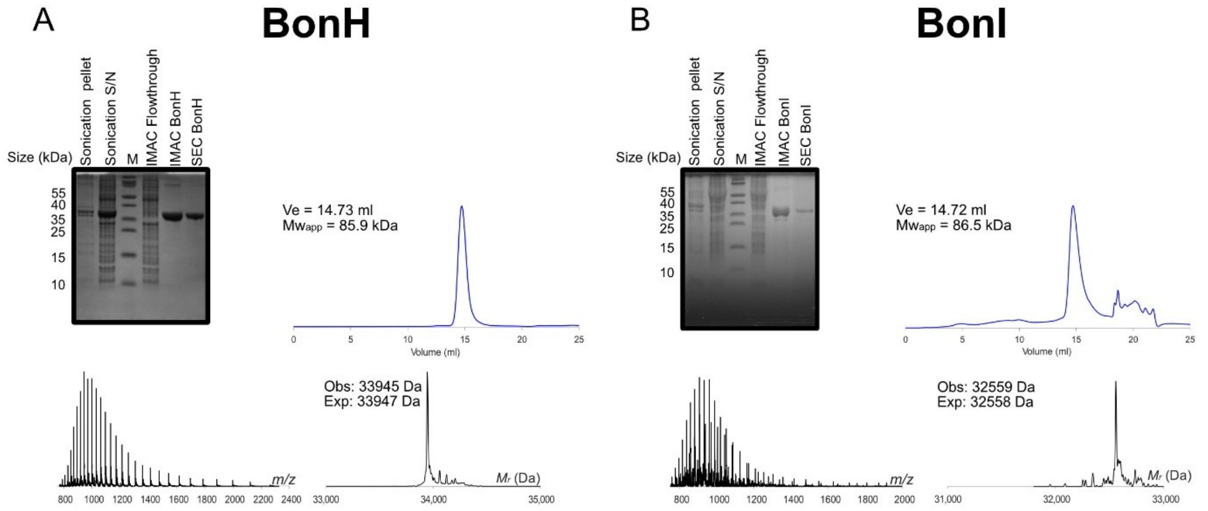

Figure S16. BonH (ECH_1) and BonI (ECH_2) purification and characterisation. A) **BonH**. SDS-PAGE following the purification after sonication, IMAC and SEC. Analytical SEC (AS200) showing His_6-BonH eluting as a trimer and non-native ESMS confirming the correct sequence of BonH. B) **BonI**. As A). SDS-PAGE following the purification after sonication, IMAC and SEC, with analytical SEC (AS200) showing elution of BonI as a trimeric species and non-native ESMS confirming the correct sequence.

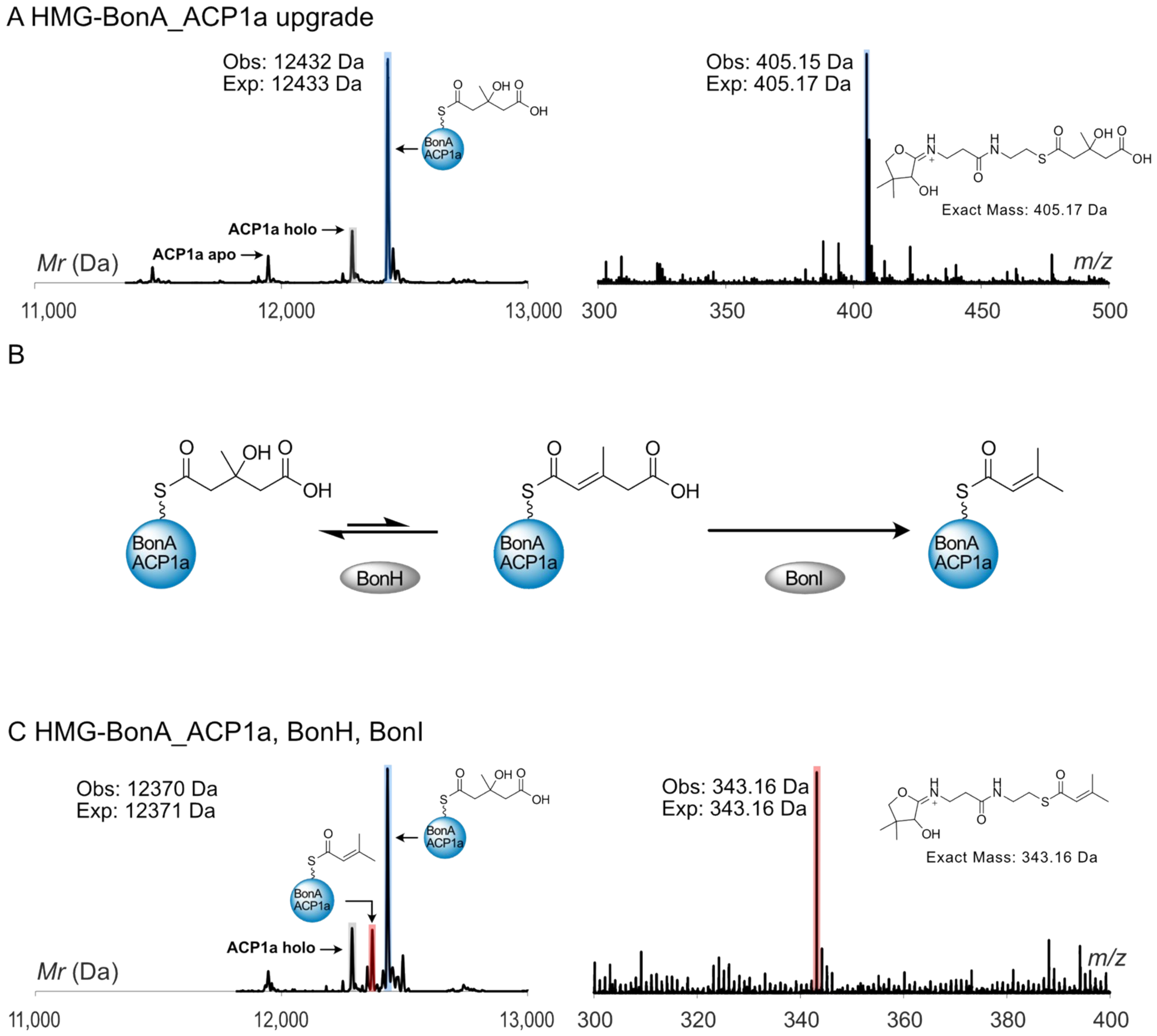

Figure S17. ECH catalysed dehydration and decarboxylation of HMG-BonA_ACP1a. A) Deconvoluted spectrum of HMG-BonA_ACP1a (obs: 12,432 Da, exp: 12,433 Da) and the Ppant ejection for this species (obs: 405.15 Da, exp: 405.17 Da), B) Proposed reaction scheme of *endo*-β-methyl-BonA_ACP1a formation via BonH and BonI. C) Deconvoluted spectrum of HMG-BonA_ACP1a when incubated with BonH and BonI to produce a *endo*-β-methyl-BonA_ACP1a species (obs: 12,370 Da, exp: 12,371 Da) and the corresponding Ppant ejection ion for this species (obs: 343.16 Da, exp: 343.16 Da).

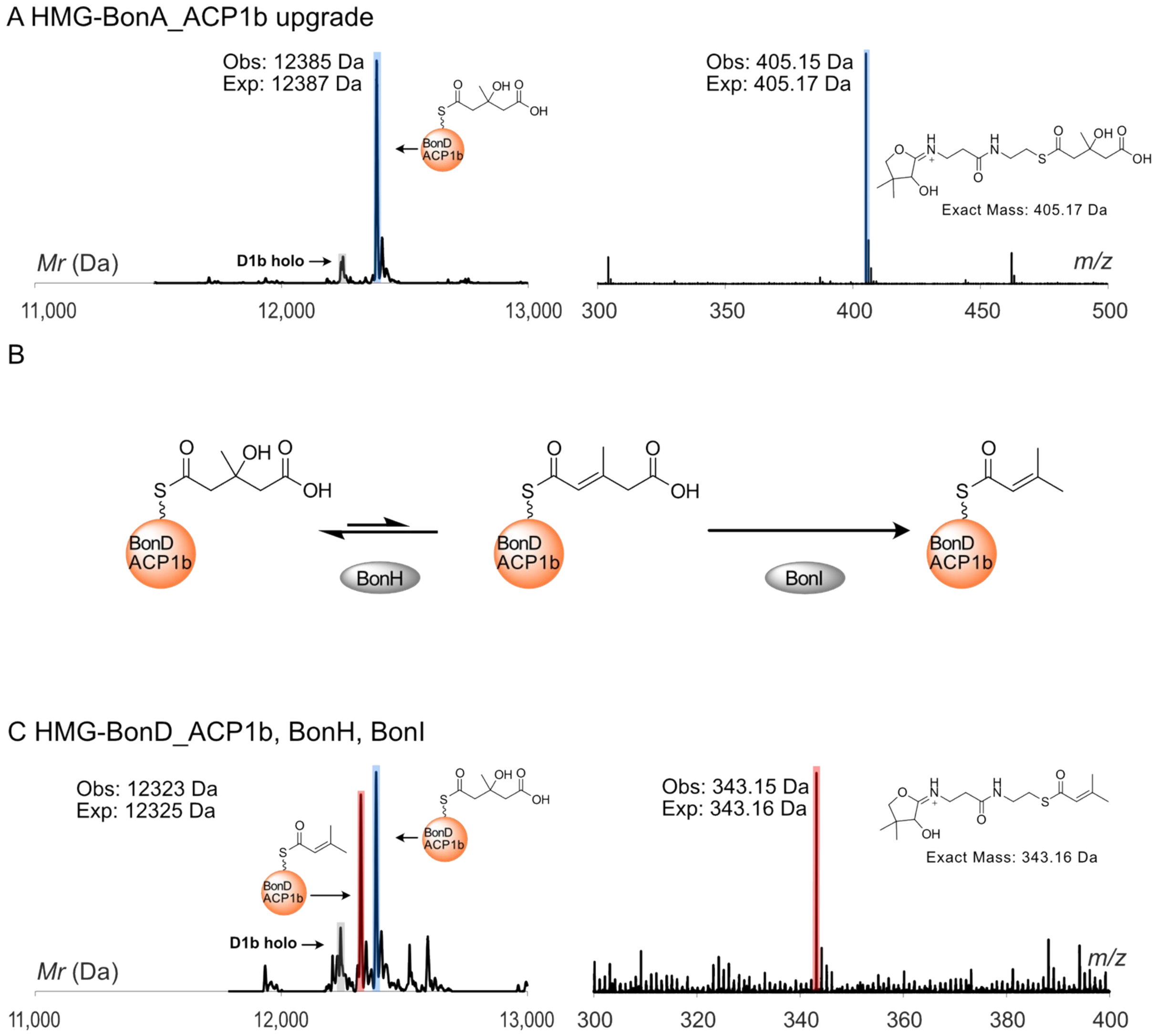

Figure S18. ECH catalysed dehydration and decarboxylation of HMG-BonD_ACP1b. A) Deconvoluted spectrum of HMG-BonD_ACP1b (obs: 12,385 Da, exp: 12,387 Da) and the Ppant ejection for this species (obs: 405.15 Da, exp: 405.17 Da), B) Proposed reaction scheme of *endo*-β-methyl-BonD_ACP1b formation via BonH and BonI. C) Deconvoluted spectrum of HMG-BonD_ACP1b when incubated with BonH and BonI to produce a *endo*-β-methyl-BonD_ACP1b species (obs: 12,323 Da, exp: 12,325 Da) and the corresponding Ppant ejection ion for this species (obs: 343.15 Da, exp: 343.16 Da).

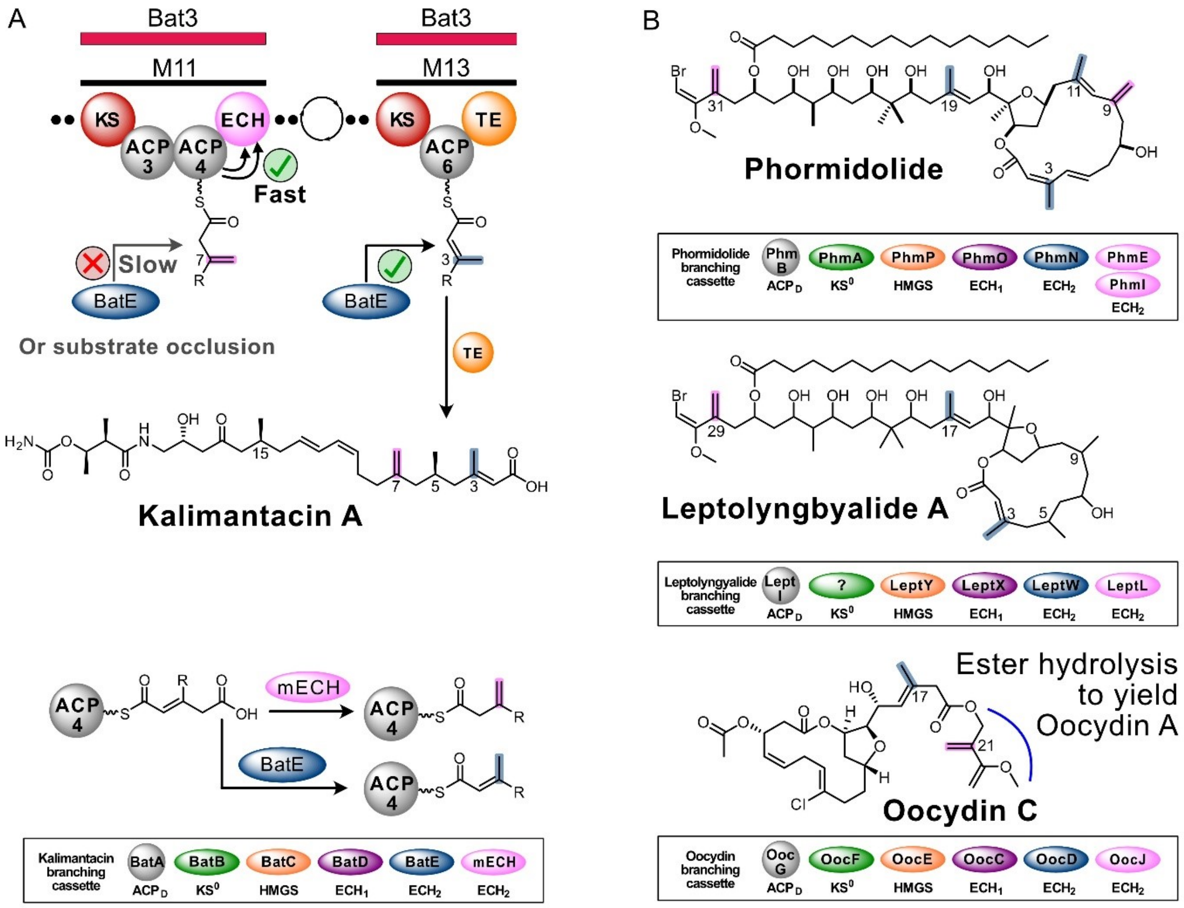

Figure S19. **Biosynthetic pathways encoding multiple ECH_2 domains**. A) β-Branch incorporation in the kalimantacin biosynthetic pathway where a *exo*-β-methylene branch (pink) is formed via a modular ECH domain (mECH) and a *endo*-β-methyl branch (blue) is formed via the *trans*-acting ECH_2 domain BatE. Both mECH and BatE domains can process a MG-ACP4 species when expressed as a discrete domain, and a mechanism of control must exist to prevent aberrant branching during biosynthesis. B) Structures of phormidolide, leptolyngbyalide A and oocydin C. Their biosynthetic pathways encode multiple ECH_2 domains and install multiple distinct branches.

List of constructs

GbnF

MHHHHHHGKPIPNPLLGLDSTENLYFQGIDPFTMNRDAIFDVLVGHTREILPELGDRQIRSEDRLADLGANSVDRAE
IVMLTLESLSLRLPLVETSGPNNLGELADLLHARLQHA

Calc Mw: 12865.56 Da

BonA_ACP1a

MHHHHHHGKPIPNPLLGLDSTENLYFQGIDPFTSLARAVRETIEQVLGEARVDSYGPHTPLMSMGLSSFDLAELRKR
LGARLGMTLDATFLLRHGTPARLLEALRET

Calc Mw: 11947.70 Da

BonA_ACP2a

MHHHHHHGKPIPNPLLGLDSTENLYFQGIDPFTALERELAASLAEALYLDSSEVDVDRPFAELGLDSIIGVEWMRAI
NRRHGLALNATLVYEHPTVRRMAARLAQAL

Calc Mw: 12000.66 Da

BonD_ACP1b

MHHHHHHGKPIPNPLLGLDSTENLYFQGIDPFTATAAARQAIRASLAATLKLDDGQLLDDQAFSSFGIDSITGVSLV
NEINERLGLALPTTVLFDYATVDQLSAHIGTLDG

Calc Mw: 11902.33 Da

BonD_ACP3b

MHHHHHHGKPIPNPLLGLDSTENLYFQGIDPFTGDLQRELAEELGEMLMLDGAAIDADTPFIDLGLDSITGVEWVR
KINGRHGLSLTAMQMYEHPTLRALAKLLAAST

Calc Mw: 11935.60 Da

BonF

MHHHHHHGKPIPNPLLGLDSTENLYFQGIDPFTMSEFSDAGVAITGVGVTSAIGQGRDAFAAALFDGAGAFAVMA
RPGRQNPAGDTRFLGAELGELAMPSGIEPRELRHVSLSARVALATLDEAWRDAALDQVDPERIALVVGGSNLQQR
EQAQVFARYAGREAFVSPAYAMSFMDTDLCGVLTRQFGIKGSACTVGGASASGQLAVIHAAQAVRSGQCDVAIAL
GALMDLSHWECQALRSLGAMGSDRFAEAPGLACRPFDLDHDGFIYGECCAALVVERIGRDAAPARGGVQPYARL
AGWHVAMDANRNPDPSLDGEKRAIDGALRAARLSAAEIDYVNPHGTGSLVGDATELAALRESGLAHARLNATKS
LVGHGLTAAGAVEVVATLVQMREHRLHPTRNLEVPIDAGFGWVGAAAQPHTIHHALTLSMGFGGINTALCLTRIH
S

Calc Mw: 47129.29 Da

BonG

MHHHHHHGKPIPNPLLGLDSTENLYFQGIDPFTMEISLMRAVGIEAMNVFGGTASLDVTSLAEHRRLDTARFNNLL
MKEKTVALPHEDPVTFAVNAARPLIDALAPEARERIEMVITCSESGIDFGKSMSTYVHHHLGLSRNCRLFEIKQACY
SGTVGLQMAINFVLAQTSPGAKVLVIATDISRYMVVEGGDALTEDWSFAEPSGGAGAVALLVGEDPRILDIDVGAN
GYYGYEVMDTCRPAPDVEAGDADLSLLSYLDCAENAFLEYQKRVGEVDYVGSFKYLAFHTPFGGMVKGAHRTM
MRKQVRAAPAAIEADFERRVMPGLQFCQRVGNIMGATVLMSIASTIANGEFDQPARIGCFSYGSGCCSEFYSGIVRA
EGRAALRGLDIGRYLDARYALSMHEYEHLLRNNSQLRFGTRNVTVRDDVVPGTLRAIPGRSQLVLREIKEFHREYE
WV

Calc Mw: 50408.48 Da

BonH

MHHHHHHGKPIPNPLLGLDSTENLYFQGIDPFTMEGGLSAIDHTAAADPAAQAWQTLRVRRQDTVCFVQFHRPEA
NNAIDHRLLSEFGAVLDMLDASVTVLVLEGLPEVFCFGADFAALREATLAGPAAAEAALEPERLYALWTRLATGPF
VVVSYVRGKANAGGLGFVAASDIVIADDSAVFSLSELLFGLMPACVMPFLVRRIGFQRAHYMTLMTQPIGVEQAA
AWGLVDAWEADGASLLRRHLLRLRRLSREALLRYKRFYSALEGSLEGDRARALAANREVFGDPANLKAIVRYVE
AGAFPWERD

Calc Mw: 33946.88 Da

BonI

MHHHHHHGKPIPNPLLGLDSTENLYFQGIDPFTMSRPAHFHGSATEMSERVVHLAELEPGIVRITLEDRTHKNAFSD
RLLNGIVEAFAEVAANPSWKAVILTGYDTYFCTGGTQDMLLKLSKGEGRFTDFPIYNLPLTCEIPVIAAMQGHAVG
GGLALGLFADFVVLSRESVYTANFMKYGFTPGFGSTLVMREKLGLPLAQALLMTAANYRGDELARHGIAFPVLPR
AEVAAHALDIARQLADKPRHSLVTLKAHLNAPLRAMLPEVTAREVEMHERTFPHPEVQQRIRALFGS

Calc Mw: 32558.49 Da

BonN

MHHHHHHGKPIPNPLLGLDSTENLYFQGIDPFTMTTREQVFETIVQHTREVVPELEGHAFQDSDSLRELGANSIDRA
EITMLVLESLSLSIPRVELFGPNNIGELADLIHARIQASVA

Calc Mw: 13209.88 Da

References

1. V. Agarwal, S. Diethelm, L. Ray, N. Garg, T. Awakawa, P. C. Dorrestein and B. S. Moore, *Org. Lett.*, 2015, **17**, 4452-4455.
2. A. J. Winter, M. T. Rowe, A. N. M. Weir, N. Akter, S. Z. Mbatha, P. D. Walker, C. Williams, Z. Song, P. R. Race, C. L. Willis and M. P. Crump, *Angew. Chem. Int. Ed.*, 2022, **61**, e202212393.
3. S. Prasad, P. B. Khadatare and I. Roy, *Appl. Environ. Microbiol.*, 2011, **77**, 4603-4609.
4. S. Tayyab, S. Qamar and M. Islam, *Biochem. Educ.*, 1991, **19**, 149-152.
5. P. D. Walker, C. Williams, A. N. M. Weir, L. Wang, J. Crosby, P. R. Race, T. J. Simpson, C. L. Willis and M. P. Crump, *Angew. Chem. Int. Ed.*, 2019, **58**, 12446-12450.
6. R. C. Edgar, *Nucleic Acids Res.*, 2004, **32**, 1792-1797.
7. K. Tamura, G. Stecher and S. Kumar, *Mol. Biol. Evol.*, 2021, **38**, 3022-3027.
8. X. Robert and P. Gouet, *Nucleic Acids Res.*, 2014, **42**, W320-W324.
9. J. Abramson, J. Adler, J. Dunger, R. Evans, T. Green, A. Pritzel, O. Ronneberger, L. Willmore, A. J. Ballard, J. Bambrick, S. W. Bodenstein, D. A. Evans, C.-C. Hung, M. O'Neill, D. Reiman, K. Tunyasuvunakool, Z. Wu, A. Žemgulytė, E. Arvaniti, C. Beattie, O. Bertolli, A. Bridgland, A. Cherepanov, M. Congreve, A. I. Cowen-Rivers, A. Cowie, M. Figurnov, F. B. Fuchs, H. Gladman, R. Jain, Y. A. Khan, C. M. R. Low, K. Perlin, A. Potapenko, P. Savy, S. Singh, A. Stecula, A. Thillaisundaram, C. Tong, S. Yakneen, E. D. Zhong, M. Zielinski, A. Žídek, V. Bapst, P. Kohli, M. Jaderberg, D. Hassabis and J. M. Jumper, *Nature*, 2024, **630**, 493-500.
